# Author Correction: Transposable elements orchestrate subgenome-convergent and -divergent transcription in common wheat

**DOI:** 10.1038/s41467-023-38754-5

**Published:** 2023-05-25

**Authors:** Yuyun Zhang, Zijuan Li, Jinyi Liu, Yu’e Zhang, Luhuan Ye, Yuan Peng, Haoyu Wang, Huishan Diao, Yu Ma, Meiyue Wang, Yilin Xie, Tengfei Tang, Yili Zhuang, Wan Teng, Yiping Tong, Wenli Zhang, Zhaobo Lang, Yongbiao Xue, Yijing Zhang

**Affiliations:** 1 National Key Laboratory of Plant Molecular Genetics, CAS Center for Excellence in Molecular Plant Sciences, Shanghai Institute of Plant Physiology and Ecology, Shanghai Institutes for Biological Sciences, Chinese Academy of Sciences, 300 Fenglin Road, Shanghai, 200032 China; 2 University of the Chinese Academy of Sciences, Beijing, 100049 China; 3 State Key Laboratory of Genetic Engineering, Collaborative Innovation Center of Genetics and Development, Department of Biochemistry, Institute of Plant Biology, School of Life Sciences, Fudan University, Shanghai, 200438 China; 4 The State Key Laboratory of Plant Cell and Chromosome Engineering, Institute of Genetics and Developmental Biology, the Innovative Academy of Seed Design, Chinese Academy of Sciences, Beijing, 100101 China; 5 Shanghai Center for Plant Stress Biology, National Key Laboratory of Plant Molecular Genetics, Center of Excellence in Molecular Plant Sciences, Shanghai Institutes for Biological Sciences, Chinese Academy of Sciences, Shanghai, 200032 China; 6 Henan University, School of Life Science, Kaifeng, Henan 457000 China; 7 State Key Laboratory for Crop Genetics and Germplasm Enhancement, Jiangsu Collaborative Innovation Center for Modern Crop Production, Nanjing Agricultural University, No.1 Weigang, Nanjing, Jiangsu 210095 China; 8 Institute of Advanced Biotechnology and School of Life Sciences, Southern University of Science and Technology, Shenzhen, 518055 China; 9 Beijing Institute of Genomics, Chinese Academy of Sciences, and National Centre for Bioinformation, Beijing, 100101 China; 10 Jiangsu Co-Innovation Center for Modern Production Technology of Grain Crops, Yangzhou University, Yangzhou, 225009 China

**Keywords:** Epigenomics, Plant evolution, Mobile elements, Regulatory networks, Transcriptional regulatory elements

Correction to: *Nature Communications* 10.1038/s41467-022-34290-w, published online 14 November 2022

The original version of the Supplementary Information associated with this Article included a truncated Supplementary Data 2 file, in which the data after row 65,537 were not included. The HTML has been updated to include a corrected version of Supplementary Data 2; the original incorrect version of Supplementary Data 2 can be found as Supplementary Information associated with this Correction.

## Supplementary information

Incorrect Supplementary Data 2

Updated Supplementary Data 2

