# Supplementary material for: Transposable elements orchestrate subgenome-convergent and -divergent transcription in common wheat
Source: Nat Commun. 2022 Nov 14;13:6940. doi: 10.1038/s41467-022-34290-w (PMC9663577; doi:10.1038/s41467-022-34290-w)
Supplement: Supplementary file 1 — Supplementary Information [file 41467_2022_34290_MOESM1_ESM.pdf]

Transposable elements orchestrate subgenome-convergent and -divergent transcription in common wheat

Zhang *et al.*

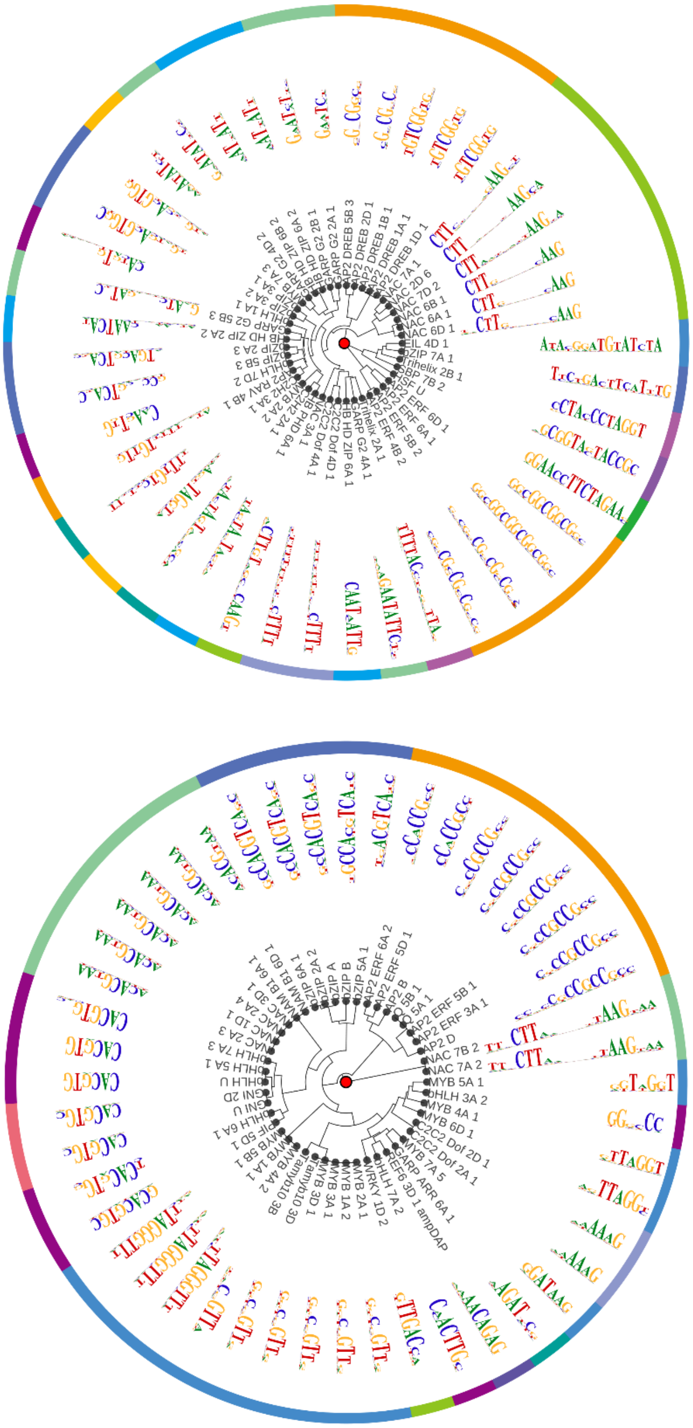

Supplementary Figure 1. Clustering of the canonical motifs *de novo* identified in high confidence TFs peaks (top) or enriched in median confidence TF peaks (bottom).

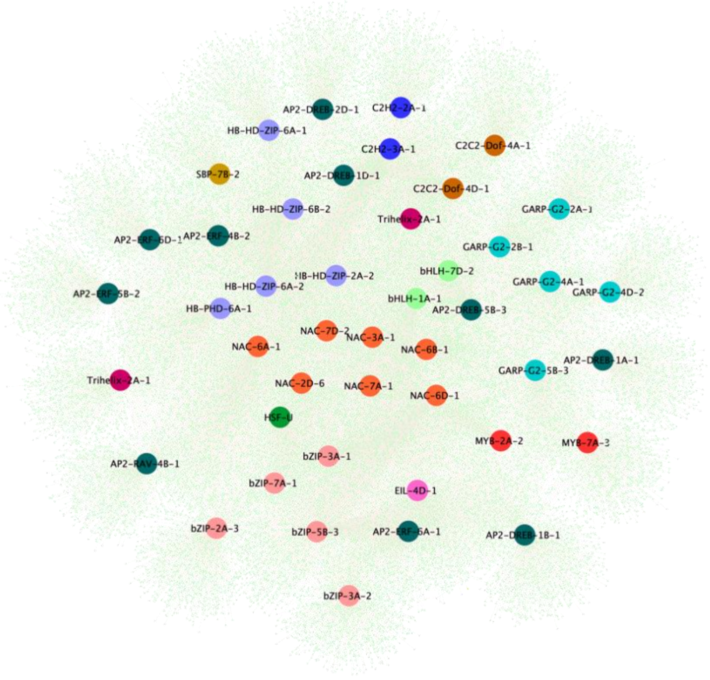

Supplementary Figure 2. Regulatory circuit integrates information from all high confidence TF–target pairs (1000 pairs for each TF were randomly selected, all targeting information listed in Supplementary Data 2). The large circle node and small circle node represent the TFs and targets, respectively. Colors of large node represent different TF families.

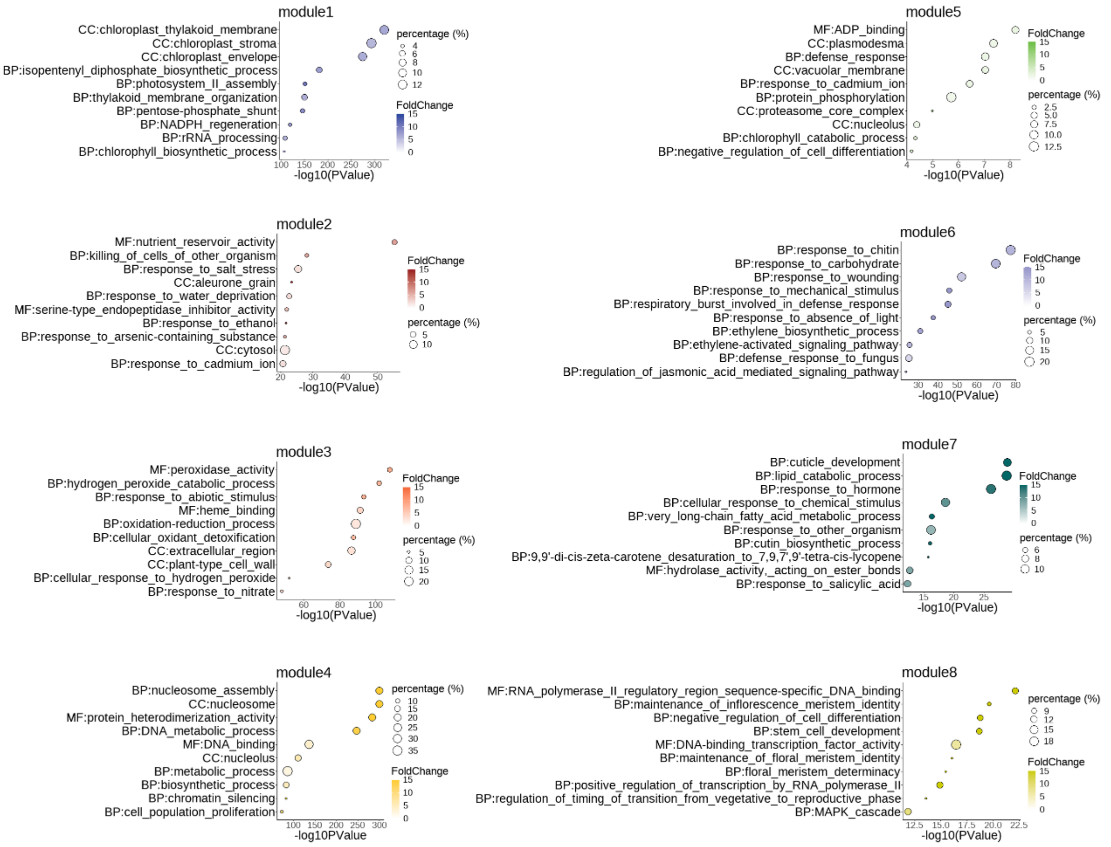

Supplementary Figure 3. GO terms top enriched for each co-expression module. Source data are provided as a Source Data file.

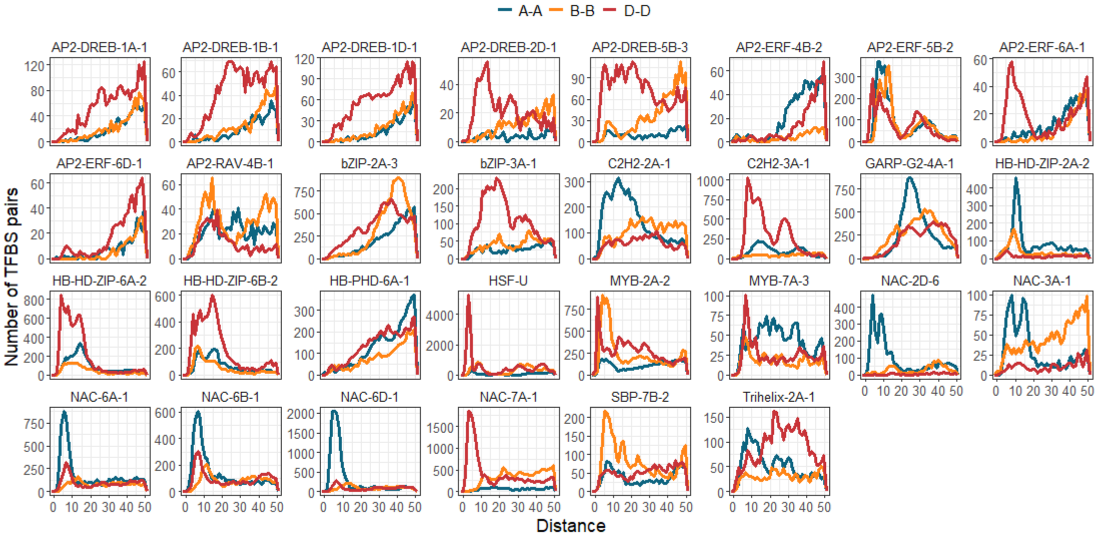

Supplementary Figure 4. K2P sequence distance distribution between subgenome-specific TFBS in each subgenome. Source data are provided as a Source Data file.

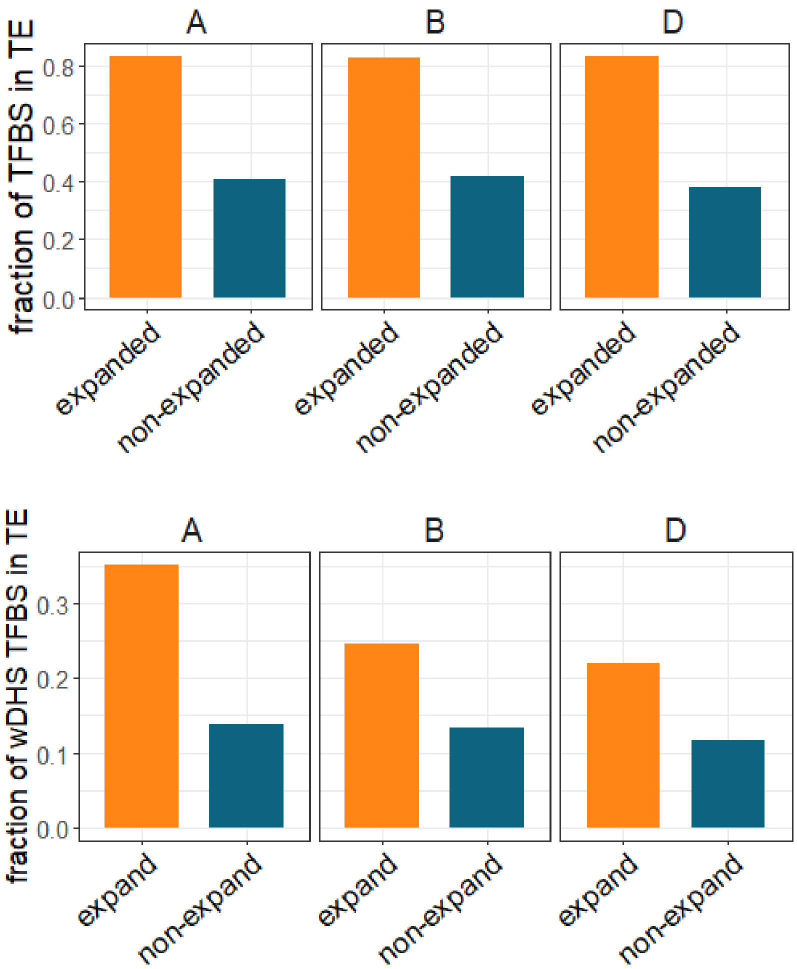

Supplementary Figure 5. Top panel, fraction of subgenome-specific TFBS embedded in TEs. TFBS pairs with similar sequences detected within subgenomes were defined as expanded TFBS and other TFBS were defined as non-expanded TFBS. BLASTN was used to align TFBS within subgenomes (see Methods). Bottom panel, fraction of subgenome-specific DHS-TFBS embedded in TEs. Expanded and non-expanded TFBS were calculated separately. Source data are provided as a Source Data file.

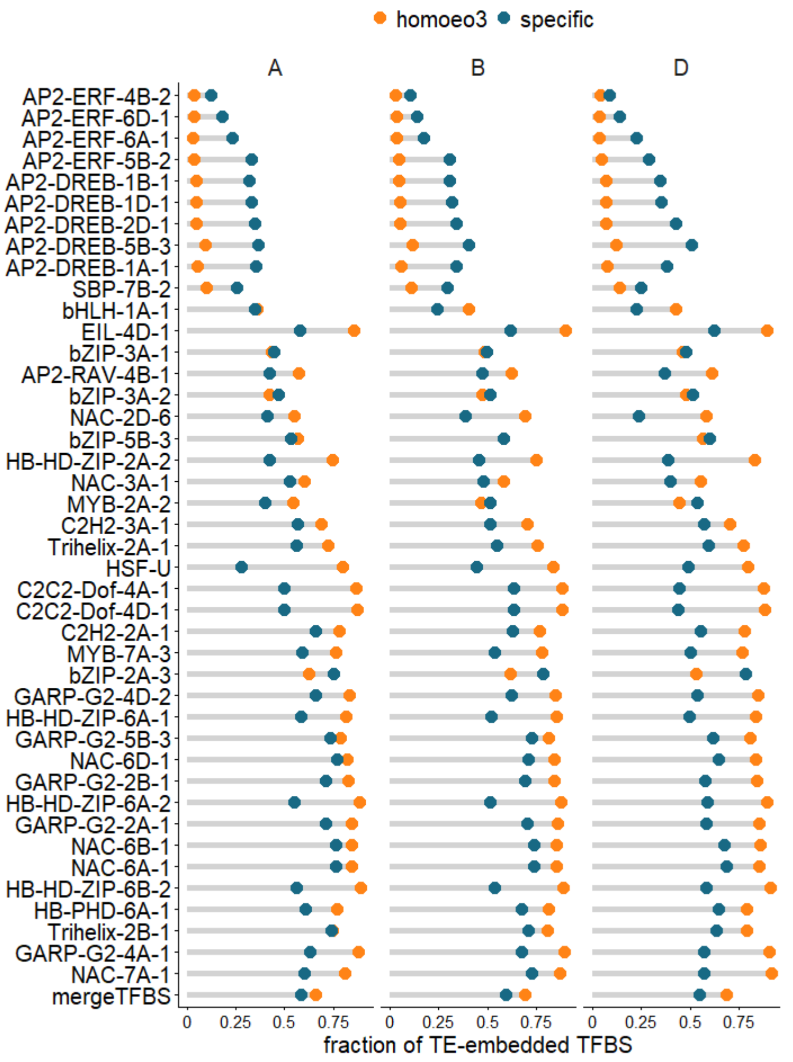

Supplementary Figure 6. Fraction of subgenome-homoeologous and subgenome-specific TFBS embedded in TEs. Shown are TFs with subgenome homoeologous and specific TFBS numbers above 100. Source data are provided as a Source Data file.

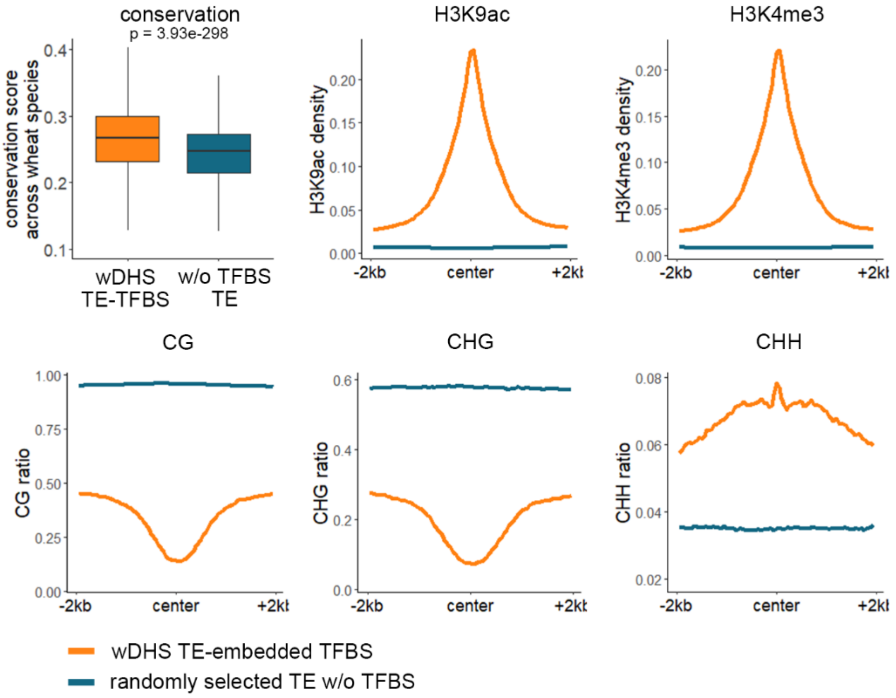

Supplementary Figure 7. Distribution of conservation score and epigenetic profiles surrounding TE-embedded TFBSs overlapping with DHS (n = 14,067), randomly selected TEs without TFBS (n = 25,287) were used as control. The average signal densities at a 50-bp resolution within a 4-kb window centered on merged TFBS centers are presented. The two-tailed Wilcoxon signed-rank test was used to compare the conservation score of different groups. Horizontal lines in boxplots show median, hinges show interquartile range (IQR), whiskers show 1.5 × IQR, points beyond 1.5 × IQR past hinge are shown. Source data are provided as a Source Data file.

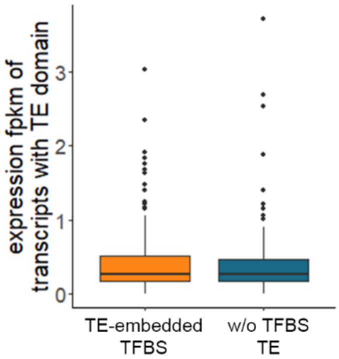

Supplementary Figure 8. Expression level of TE transcripts containing transposition related domains overlapping with TFBS (n = 248), randomly selected TEs without TFBS (n = 159) were used as control. Horizontal lines in boxplots show median, hinges show IQR, whiskers show 1.5 × IQR, points beyond 1.5 × IQR past hinge are shown. Source data are provided as a Source Data file.

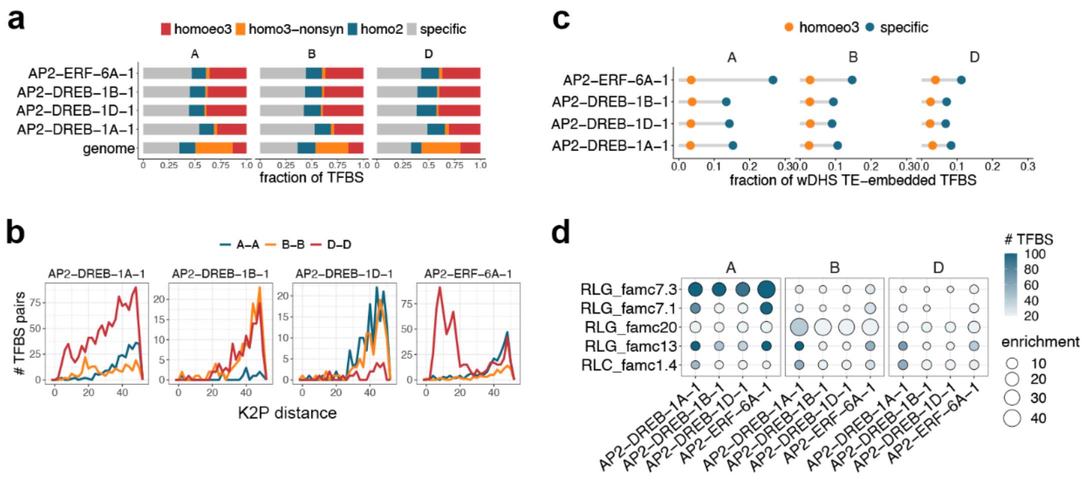

Supplementary Figure 9. TE contributed to TFBS expansion (results obtained using replicated DAP-seq data). a, Fraction of TFBSs in subgenome-homologous regions as determined by the reciprocal alignment between subgenomes. **b**, K2P sequence distance distribution between subgenome-specific TFBS in each subgenome. **c**, Fraction of subgenome-homoeologous and -specific TFBSs embedded in TEs within open chromatin regions characterized by DHS. **d**, Enriched TE subfamilies harboring subgenome-specific TFBSs, with the fraction of each TE subfamily across the genome as the background. Source data are provided as a Source Data file.

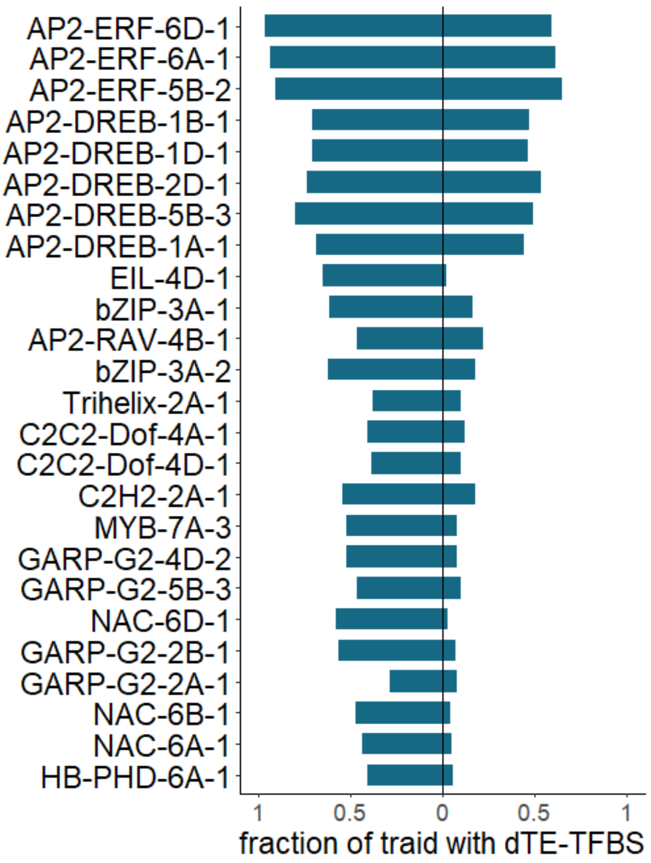

Supplementary Figure 10. Fractions of targeted triads with degenerated TE-derived TFBS in balanced and unbalanced triads. Source data are provided as a Source Data file.

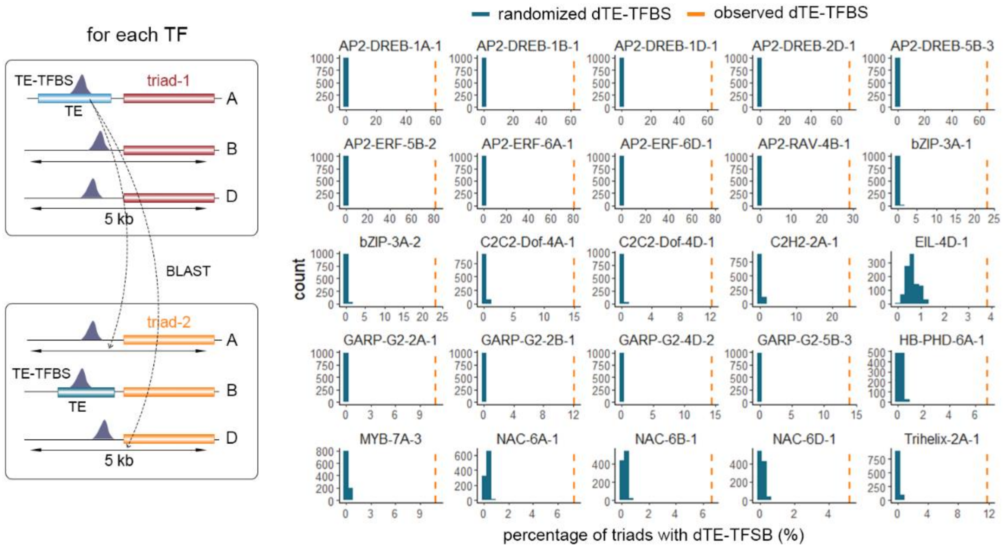

Supplementary Figure 11. Permutation test of the significance of defined dTEs. Left panel: sequence comparison between TEs contributed to TFBS and 5 kb region centered on transcription start site of different triad genes. The aligned region with TFBS was defined as randomized dTE-TFBS. Right panel: fraction of triads with dTE-TFBS. Blue bar is the distribution of fraction of triads with randomized dTE-TFBS in 1,000 times sequence alignment. Yellow line is the fraction of triads with observed dTE-TFBS (Fig. 6d). Source data are provided as a Source Data file.

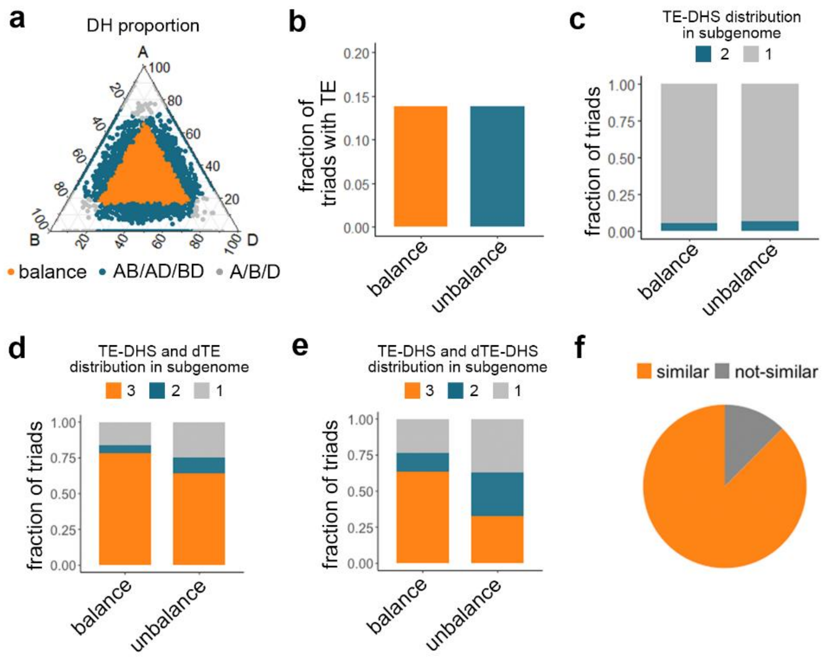

Supplementary Figure 12. The impact of TEs on *in vivo* subgenome-convergent regulation. a, Illustration of the quantitative divergence of chromatin openness in triad promoters. Orange dots represent subgenome-balanced DHS level. Blue and grey dos represent subgenome-unbalanced DHS levels. **b**, Fraction of triads with balanced and unbalanced DH density at TE-embedded DHS. **c**, Fraction of triads with balanced and unbalanced DH density at TE-embedded DHS present in one, two or three subgenomes. **d**, Fraction of triads with balanced and unbalanced DH density at TE-embeded DHS and corresponding dTE in one, two or three subgenomes. **e**, Fraction of triads with balanced and unbalanced DH density with TE- and dTE-embedded DHS present in one, two or three subgenomes. **f**, Fraction of triads with high sequence similarity between TE-embedded DHS and dTE-derived DHS. Source data are provided as a Source Data file.

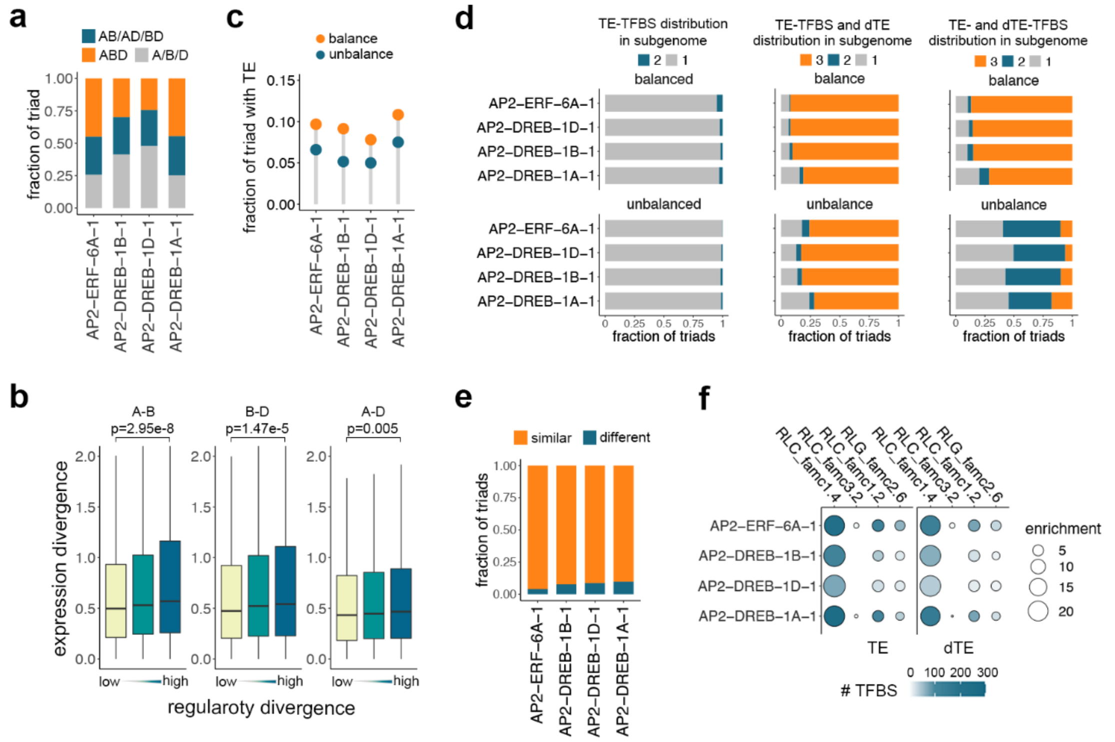

Supplementary Figure 13. TE contribution to subgenome-convergent regulation (results obtained using replicated DAP-seq data). a, Fraction of balanced and unbalanced TF binding in the promoters of triad genes (1:1:1 correspondence across subgenomes). **b**, Correlation of subgenome-biased binding and expression. Homoeolog pairs of triads were grouped according to TF binding divergence, and the two-tailed Wilcoxon signed-rank test was used to compare expression divergence between subgenomes A and B (n = 7,057), B and D (n = 5,487), A and D (n = 7,180), respectively. Horizontal lines in boxplots show median, hinges show IQR, whiskers show 1.5 × IQR, points beyond 1.5 × IQR past hinge are shown. **c**, Fraction of triads with balanced and unbalanced TF binding embedded in TEs. **d**, Left: fraction of triads with balanced and unbalanced TF binding embedded in TEs present in one, two or three subgenomes. Middle: fraction of triads with balanced and unbalanced TF binding embedded in TE-embedded TFBS and corresponding dTE-derived TFBS present in one, two or three subgenomes. Right: fraction of balanced and unbalanced triads with TE-embedded and dTE-derived TFBS present in one, two or three subgenomes. **e**, Fraction of triads with high sequence similarity of TFBS derived from TE and corresponding dTE. **f**, Enrichment of TE (left) and dTE (right) families that contributed to the balanced TF binding across triad promoters, with the fraction of TE in the genome as the background. Source data are provided as a Source Data file.

a

TE	# motifs
RLC_famc1.4	19,842,288
non RLC_famc1.4	25,849,421

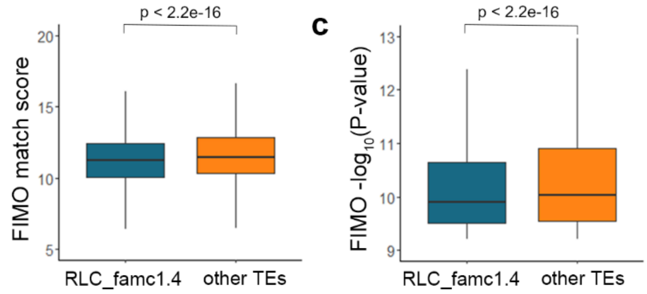

Supplementary Figure 14. The occurrence and quality of motifs from JASPAR database in RLC_famc1.4 and randomly selected non-RLC_famc1.4 TE copies with the same total length. a, The number of motifs in RLC_famc1.4 and non-RLC_famc1.4 TE copies. **b-c,** The match score and significance of motifs in RLC_famc1.4 (n = 19,842,288) and non-RLC_famc1.4 (n = 25,849,421) TE copies. The two-tailed Wilcoxon signed-rank test was used to compare the match score and significance of RLC_famc1.4 and non-RLC_famc1.4. Horizontal lines in boxplots show median, hinges show IQR, whiskers show 1.5 × IQR, points beyond 1.5 × IQR past hinge are shown. Source data are provided as a Source Data file.
