# Supplementary material for: Transposable elements orchestrate subgenome-convergent and -divergent transcription in common wheat
Source: Nat Commun. 2022 Nov 14;13:6940. doi: 10.1038/s41467-022-34290-w (PMC9663577; doi:10.1038/s41467-022-34290-w)
Supplement: Supplementary file 3 — Description of Additional Supplementary Files [file 41467_2022_34290_MOESM3_ESM.pdf]

Description of Additional Supplementary Files

File Name: Supplementary Data 1
Description: Information of TFs whose bindings were profiled by DAP-seq. The longest isoform was selected for DAP assay.

File Name: Supplementary Data 2
Description: List of HC TF target genes.

File Name: Supplementary Data 3
Description: The homeologous relationship of 189 TFs.
