# Supplementary material for: Transposable elements orchestrate subgenome-convergent and -divergent transcription in common wheat
Source: Nat Commun. 2022 Nov 14;13:6940. doi: 10.1038/s41467-022-34290-w (PMC9663577; doi:10.1038/s41467-022-34290-w)
Supplement: Supplementary file 7 — Reporting Summary [file 41467_2022_34290_MOESM7_ESM.pdf]

nature portfolio

Corresponding author(s): Yijing Zhang, Yongbiao Xue, Zhaobo Lang

Last updated by author(s): Oct 9, 2022

Reporting Summary

Nature Portfolio wishes to improve the reproducibility of the work that we publish. This form provides structure for consistency and transparency in reporting. For further information on Nature Portfolio policies, see our Editorial Policies and the Editorial Policy Checklist.

Statistics

For all statistical analyses, confirm that the following items are present in the figure legend, table legend, main text, or Methods section.

n/a	Confirmed	
☐	☒	The exact sample size (*n*) for each experimental group/condition, given as a discrete number and unit of measurement
☒	☐	A statement on whether measurements were taken from distinct samples or whether the same sample was measured repeatedly
☐	☒	The statistical test(s) used AND whether they are one- or two-sided<br>*Only common tests should be described solely by name; describe more complex techniques in the Methods section.*
☒	☐	A description of all covariates tested
☒	☐	A description of any assumptions or corrections, such as tests of normality and adjustment for multiple comparisons
☐	☒	A full description of the statistical parameters including central tendency (e.g. means) or other basic estimates (e.g. regression coefficient) AND variation (e.g. standard deviation) or associated estimates of uncertainty (e.g. confidence intervals)
☐	☒	For null hypothesis testing, the test statistic (e.g. *F*, *t*, *r*) with confidence intervals, effect sizes, degrees of freedom and *P* value noted<br>*Give P values as exact values whenever suitable.*
☒	☐	For Bayesian analysis, information on the choice of priors and Markov chain Monte Carlo settings
☒	☐	For hierarchical and complex designs, identification of the appropriate level for tests and full reporting of outcomes
☐	☒	Estimates of effect sizes (e.g. Cohen's *d*, Pearson's *r*), indicating how they were calculated

Our web collection on statistics for biologists contains articles on many of the points above.

Software and code

Policy information about availability of computer code

Data collection	TF DAP-seq libraries were sequenced with Illumina NovaSeq 6000 system.
Data analysis	fastp (version 0.20.0); Trim Galore (version 0.4.4); Burrows–Wheeler Aligner (version 0.7.17-r1188); HISAT2 (version 2.2.1); MACS (version 2.2.6); featureCount program of the Subread package (version 2.0.0); HiC-Pro (version 2.11.1); Homer (version 4.11); Juicer (version version 1.21.01); Juicerbox (version 1.11.08); MEME software toolkit (version 5.1.1); R package motifStack (version 1.34.0); R package universalmotif (version 1.4.0); mummer (version 4.0.0beta2); ROAST (version 3) from multiz; PHAST(version 1.4); R package WGCNA (version 1.70.3); Cytoscape (version 3.8.2); BLASTN (version 2.9.0); Circos(version 0.69-8); MAFFT (version v7.149b); EMBOSS (version 6.6.0.0); FastTree (version 2.1.10); R package ggtree (version 2.4.1); Orthofinder(version 2.3.12); Jalview (version 2.11.1.3); Integrative Genomics Viewer (version 2.8.10)

For manuscripts utilizing custom algorithms or software that are central to the research but not yet described in published literature, software must be made available to editors and reviewers. We strongly encourage code deposition in a community repository (e.g. GitHub). See the Nature Portfolio guidelines for submitting code & software for further information.

Data

Policy information about availability of data

All manuscripts must include a data availability statement. This statement should provide the following information, where applicable:

- Accession codes, unique identifiers, or web links for publicly available datasets
- A description of any restrictions on data availability
- For clinical datasets or third party data, please ensure that the statement adheres to our policy

The DAP-sequencing data generated in this study have been submitted to the NCBI Gene Expression Omnibus (GEO; https://www.ncbi.nlm.nih.gov/geo/) under

accession number GSE192815 (https://www.ncbi.nlm.nih.gov/geo/GSE192815). Tracks for all sequencing data can be visualized through our local genome browser (http://bioinfo.sibs.ac.cn/dap-seq_CS_jbrowse/). histone ChIP-seq data of 7 typical tissues and 8 external stimuli, RNA-seq and DNase-seq data of Chinese Spring(CS) seedling are under accession numbers GSE139019 and GSE121903 in NCBI GEO database. Hi-C data of CS is under accession number GSE133885 deposited in NCBI GEO database. The hexaploid wheat transcriptomic data of 536 samples were downloaded from Wheat Expression Browser (http://www.wheat-expression.com/). The OsHOX24 ChIP-seq data of Oryza sativa is under accession number GSE144419 deposited in NCBI GEO database. The TFBS of Arabidopsis thaliana were downloaded from Plant Cistrome Database (http://neomorph.salk.edu/dap_web/pages/index.php). Scripts are available at https://github.com/yuyun-zhang/hexa_dap.

Field-specific reporting

Please select the one below that is the best fit for your research. If you are not sure, read the appropriate sections before making your selection.

- [x] Life sciences
- [ ] Behavioural & social sciences
- [ ] Ecological, evolutionary & environmental sciences

For a reference copy of the document with all sections, see nature.com/documents/nr-reporting-summary-flat.pdf

Life sciences study design

All studies must disclose on these points even when the disclosure is negative.

Sample size: No statistics method is used to predetermine sample size for TF DAP-seq. We cloned 189 TFs from 30 families, of which 107 were highly expressed TFs and 82 were functionally annotated TFs or hub TFs in the co-expression network. The 30 TF families (63% of all large TF families with the number of TFs > 100 in CS) is the sufficient sample size to analyze the regulatory pattern of subgenomes.

Data exclusions: The TF data were classified according to the de novo identification or enrichment of canonical representative motifs, resulting in 45 high-, 48 median-, and 97 low-confidence TF datasets (HC, MC and LC) (Fig. 1a and Fig. S1). The HC and MC were used for the main analysis.

Replication: Replicated assays were performed and reproduced the main results in this study (Fig. S9 and Fig. S13).

Randomization: Because we did not have group allocation and perform different treatments in this study, the randomization was not used.

Blinding: Because we did not have group allocation in this study, the blinding was not used.

Reporting for specific materials, systems and methods

We require information from authors about some types of materials, experimental systems and methods used in many studies. Here, indicate whether each material, system or method listed is relevant to your study. If you are not sure if a list item applies to your research, read the appropriate section before selecting a response.

Materials & experimental systems

n/a	Involved in the study
[ ]	[x] Antibodies
[x]	[ ] Eukaryotic cell lines
[x]	[ ] Palaeontology and archaeology
[x]	[ ] Animals and other organisms
[x]	[ ] Human research participants
[x]	[ ] Clinical data
[x]	[ ] Dual use research of concern

Methods

n/a	Involved in the study
[ ]	[x] ChIP-seq
[x]	[ ] Flow cytometry
[x]	[ ] MRI-based neuroimaging

Antibodies

Antibodies used: Halo: Promega, cat # G7281, lot # 0000384548

Validation: The Halo antibody has been validated by the supplier. The reports could be found on the the supplier website.

ChIP-seq

Data deposition

- [x] Confirm that both raw and final processed data have been deposited in a public database such as GEO.
- [x] Confirm that you have deposited or provided access to graph files (e.g. BED files) for the called peaks.

Data access links
May remain private before publication.: https://www.ncbi.nlm.nih.gov/geo/query/acc.cgi?acc=GSE192815

Files in database submission

Raw data: Alfin-4D-1_1.fq.gz;Alfin-4D-1_2.fq.gz;AP2-B_1.fq.gz;AP2-B_2.fq.gz;AP2-D_1.fq.gz;AP2-D_2.fq.gz;AP2-DREB-1A-1_1.fq.gz;AP2-DREB-1A-1_2.fq.gz;AP2-DREB-1B-1_1.fq.gz;AP2-DREB-1B-1_2.fq.gz;AP2-DREB-1D-1_1.fq.gz;AP2-DREB-1D-1_2.fq.gz;AP2-DREB-2D-1_1.fq.gz;AP2-DREB-2D-1_2.fq.gz;AP2-DREB-5B-3_1.fq.gz;AP2-DREB-5B-3_2.fq.gz;AP2-ERF-3A-1_1.fq.gz;AP2-ERF-3A-1_2.fq.gz;AP2-ERF-4B-2_1.fq.gz;AP2-ERF-4B-2_2.fq.gz;AP2-ERF-5B-1_1.fq.gz;AP2-ERF-5B-1_2.fq.gz;AP2-ERF-5B-2_1.fq.gz;AP2-ERF-5B-2_2.fq.gz;AP2-ERF-5D-1_1.fq.gz;AP2-ERF-5D-1_2.fq.gz;AP2-ERF-6A-1_1.fq.gz;AP2-ERF-6A-1_2.fq.gz;AP2-ERF-6A-2_1.fq.gz;AP2-ERF-6A-2_2.fq.gz;AP2-ERF-6D-1_1.fq.gz;AP2-ERF-6D-1_2.fq.gz;AP2-RAV-4B-1_1.fq.gz;AP2-RAV-4B-1_2.fq.gz;B3-2A-1_1.fq.gz;B3-2A-1_2.fq.gz;B3-2B-2_1.fq.gz;B3-2B-2_2.fq.gz;B3-4A-1_1.fq.gz;B3-4A-1_2.fq.gz;B3-4B-1_1.fq.gz;B3-4B-1_2.fq.gz;B3-4B-2_1.fq.gz;B3-4B-2_2.fq.gz;B3-5A-1_1.fq.gz;B3-5A-1_2.fq.gz;B3-5B-1_1.fq.gz;B3-5B-1_2.fq.gz;B3-5D-2_1.fq.gz;B3-5D-2_2.fq.gz;B3-6D-1_1.fq.gz;B3-6D-1_2.fq.gz;B3-6D-2_1.fq.gz;B3-6D-2_2.fq.gz;B3-7B-1_1.fq.gz;B3-7B-1_2.fq.gz;B3-7D-1_1.fq.gz;B3-7D-1_2.fq.gz;B3-ARF-2D-1_1.fq.gz;B3-ARF-2D-1_2.fq.gz;B3-ARF-3A-1_1.fq.gz;B3-ARF-3A-1_2.fq.gz;B3-ARF-3B-2_1.fq.gz;B3-ARF-3B-2_2.fq.gz;BES1-6A-1_1.fq.gz;BES1-6A-1_2.fq.gz;BES1-A_1.fq.gz;BES1-A_2.fq.gz;BES1-B_1.fq.gz;BES1-B_2.fq.gz;BES1-D_1.fq.gz;BES1-D_2.fq.gz;bHLH-1A-1_1.fq.gz;bHLH-1A-1_2.fq.gz;bHLH-3A-2_1.fq.gz;bHLH-3A-2_2.fq.gz;bHLH-4B-1_1.fq.gz;bHLH-4B-1_2.fq.gz;bHLH-5A-1_1.fq.gz;bHLH-5A-1_2.fq.gz;bHLH-5A-4_1.fq.gz;bHLH-5A-4_2.fq.gz;bHLH-6A-1_1.fq.gz;bHLH-6A-1_2.fq.gz;bHLH-7A-2_1.fq.gz;bHLH-7A-2_2.fq.gz;bHLH-7A-3_1.fq.gz;bHLH-7A-3_2.fq.gz;bHLH-7D-2_1.fq.gz;bHLH-7D-2_2.fq.gz;bHLH-U_1.fq.gz;bHLH-U_2.fq.gz;bZIP-2A-2_1.fq.gz;bZIP-2A-2_2.fq.gz;bZIP-2A-3_1.fq.gz;bZIP-2A-3_2.fq.gz;bZIP-3A-1_1.fq.gz;bZIP-3A-1_2.fq.gz;bZIP-3A-2_1.fq.gz;bZIP-3A-2_2.fq.gz;bZIP-5A-1_1.fq.gz;bZIP-5A-1_2.fq.gz;bZIP-5B-3_1.fq.gz;bZIP-5B-3_2.fq.gz;bZIP-6A-1_1.fq.gz;bZIP-6A-1_2.fq.gz;bZIP-7A-1_1.fq.gz;bZIP-7A-1_2.fq.gz;bZIP-A_1.fq.gz;bZIP-A_2.fq.gz;bZIP-B_1.fq.gz;bZIP-B_2.fq.gz;bZIP-D_1.fq.gz;bZIP-D_2.fq.gz;C2-A_1.fq.gz;C2-A_2.fq.gz;C2-B_1.fq.gz;C2-B_2.fq.gz;C2C2-Dof-2A-1_1.fq.gz;C2C2-Dof-2A-1_2.fq.gz;C2C2-Dof-2D-1_1.fq.gz;C2C2-Dof-2D-1_2.fq.gz;C2C2-Dof-4A-1_1.fq.gz;C2C2-Dof-4A-1_2.fq.gz;C2C2-Dof-4D-1_1.fq.gz;C2C2-Dof-4D-1_2.fq.gz;C2-D_1.fq.gz;C2-D_2.fq.gz;C2H2-2A-1_1.fq.gz;C2H2-2A-1_2.fq.gz;C2H2-3A-1_1.fq.gz;C2H2-3A-1_2.fq.gz;C2H2-3D-2_1.fq.gz;C2H2-3D-2_2.fq.gz;C2H2-4A-1_1.fq.gz;C2H2-4A-1_2.fq.gz;C2H2-5B-1_1.fq.gz;C2H2-5B-1_2.fq.gz;C2H2-6A-1_1.fq.gz;C2H2-6A-1_2.fq.gz;C2H2-6B-1_1.fq.gz;C2H2-6B-1_2.fq.gz;C2H2-6D-2_1.fq.gz;C2H2-6D-2_2.fq.gz;C3H-1A-1_1.fq.gz;C3H-1A-1_2.fq.gz;C3H-1B-1_1.fq.gz;C3H-1B-1_2.fq.gz;C3H-1D-2_1.fq.gz;C3H-1D-2_2.fq.gz;C3H-3A-1_1.fq.gz;C3H-3A-1_2.fq.gz;C3H-3A-2_1.fq.gz;C3H-3A-2_2.fq.gz;C3H-4B-1_1.fq.gz;C3H-4B-1_2.fq.gz;C3H-7B-1_1.fq.gz;C3H-7B-1_2.fq.gz;DBB-7A-1_1.fq.gz;DBB-7A-1_2.fq.gz;DBP-1B-1_1.fq.gz;DBP-1B-1_2.fq.gz;DBP-1D-1_1.fq.gz;DBP-1D-1_2.fq.gz;EIL-4D-1_1.fq.gz;EIL-4D-1_2.fq.gz;GARP-ARR-6A-1_1.fq.gz;GARP-ARR-6A-1_2.fq.gz;GARP-ARR-7A-1_1.fq.gz;GARP-ARR-7A-1_2.fq.gz;GARP-ARR-7A-3_1.fq.gz;GARP-ARR-7A-3_2.fq.gz;GARP-G2-2A-1_1.fq.gz;GARP-G2-2A-1_2.fq.gz;GARP-G2-2B-1_1.fq.gz;GARP-G2-2B-1_2.fq.gz;GARP-G2-3B-1_1.fq.gz;GARP-G2-3B-1_2.fq.gz;GARP-G2-4A-1_1.fq.gz;GARP-G2-4A-1_2.fq.gz;GARP-G2-4D-2_1.fq.gz;GARP-G2-4D-2_2.fq.gz;GARP-G2-5B-3_1.fq.gz;GARP-G2-5B-3_2.fq.gz;GRAS-2A-1_1.fq.gz;GRAS-2A-1_2.fq.gz;GRAS-4B-1_1.fq.gz;GRAS-4B-1_2.fq.gz;GRAS-4B-2_1.fq.gz;GRAS-4B-2_2.fq.gz;GRF4-2A-1_1.fq.gz;GRF4-2A-1_2.fq.gz;GRF4-2B-1_1.fq.gz;GRF4-2B-1_2.fq.gz;GRF4-2D-1_1.fq.gz;GRF4-2D-1_2.fq.gz;GRF-6A-1_1.fq.gz;GRF-6A-1_2.fq.gz;HB-BELL-4B-1_1.fq.gz;HB-BELL-4B-1_2.fq.gz;HB-BELL-5D-1_1.fq.gz;HB-BELL-5D-1_2.fq.gz;HB-HD-ZIP-2A-1_1.fq.gz;HB-HD-ZIP-2A-1_2.fq.gz;HB-HD-ZIP-2A-2_1.fq.gz;HB-HD-ZIP-2A-2_2.fq.gz;HB-HD-ZIP-2B-1_1.fq.gz;HB-HD-ZIP-2B-1_2.fq.gz;HB-HD-ZIP-6A-1_1.fq.gz;HB-HD-ZIP-6A-1_2.fq.gz;HB-HD-ZIP-6A-2_1.fq.gz;HB-HD-ZIP-6A-2_2.fq.gz;HB-HD-ZIP-6B-2_1.fq.gz;HB-HD-ZIP-6B-2_2.fq.gz;HB-PHD-6A-1_1.fq.gz;HB-PHD-6A-1_2.fq.gz;HB-WOX-A_1.fq.gz;HB-WOX-A_2.fq.gz;HB-WOX-B_1.fq.gz;HB-WOX-B_2.fq.gz;HB-WOX-D_1.fq.gz;HB-WOX-D_2.fq.gz;HSF-U_1.fq.gz;HSF-U_2.fq.gz;IPA1-7B-1_1.fq.gz;IPA1-7B-1_2.fq.gz;LIM-7B-1_1.fq.gz;LIM-7B-1_2.fq.gz;LIM-7D-1_1.fq.gz;LIM-7D-1_2.fq.gz;LOB-1A-1_1.fq.gz;LOB-1A-1_2.fq.gz;LOB-3A-1_1.fq.gz;LOB-3A-1_2.fq.gz;LOB-3B-1_1.fq.gz;LOB-3B-1_2.fq.gz;LOB-3D-1_1.fq.gz;LOB-3D-1_2.fq.gz;LOB-3D-2_1.fq.gz;LOB-3D-2_2.fq.gz;MADS1-lgy3-4A-1_1.fq.gz;MADS1-lgy3-4A-1_2.fq.gz;MADS1-lgy3-4B-1_1.fq.gz;MADS1-lgy3-4B-1_2.fq.gz;MADS1-lgy3-4D-1_1.fq.gz;MADS1-lgy3-4D-1_2.fq.gz;MADS-M-4D-1_1.fq.gz;MADS-M-4D-1_2.fq.gz;MADS-MIKC-5A-1_1.fq.gz;MADS-MIKC-5A-1_2.fq.gz;MADS-MIKC-7A-1_1.fq.gz;MADS-MIKC-7A-1_2.fq.gz;MYB-1A-1_1.fq.gz;MYB-1A-1_2.fq.gz;MYB-1A-2_1.fq.gz;MYB-1A-2_2.fq.gz;MYB-2A-1_1.fq.gz;MYB-2A-1_2.fq.gz;MYB-2A-2_1.fq.gz;MYB-2A-2_2.fq.gz;MYB-3A-1_1.fq.gz;MYB-3A-1_2.fq.gz;MYB-3D-1_1.fq.gz;MYB-3D-1_2.fq.gz;MYB-4A-1_1.fq.gz;MYB-4A-1_2.fq.gz;MYB-4A-2_1.fq.gz;MYB-4A-2_2.fq.gz;MYB-5A-1_1.fq.gz;MYB-5A-1_2.fq.gz;MYB-5B-1_1.fq.gz;MYB-5B-1_2.fq.gz;MYB-6D-1_1.fq.gz;MYB-6D-1_2.fq.gz;MYB-7A-3_1.fq.gz;MYB-7A-3_2.fq.gz;MYB-7A-5_1.fq.gz;MYB-7A-5_2.fq.gz;NAC-1D-1_1.fq.gz;NAC-1D-1_2.fq.gz;NAC-2A-1_1.fq.gz;NAC-2A-1_2.fq.gz;NAC-2A-3_1.fq.gz;NAC-2A-3_2.fq.gz;NAC-2A-4_1.fq.gz;NAC-2A-4_2.fq.gz;NAC-2D-6_1.fq.gz;NAC-2D-6_2.fq.gz;NAC-3A-1_1.fq.gz;NAC-3A-1_2.fq.gz;NAC-3D-1_1.fq.gz;NAC-3D-1_2.fq.gz;NAC-6A-1_1.fq.gz;NAC-6A-1_2.fq.gz;NAC-6B-1_1.fq.gz;NAC-6B-1_2.fq.gz;NAC-6D-1_1.fq.gz;NAC-6D-1_2.fq.gz;NAC-7A-1_1.fq.gz;NAC-7A-1_2.fq.gz;NAC-7A-2_1.fq.gz;NAC-7A-2_2.fq.gz;NAC-7B-2_1.fq.gz;NAC-7B-2_2.fq.gz;NAC-7D-2_1.fq.gz;NAC-7D-2_2.fq.gz;NAM-B1-6A-1_1.fq.gz;NAM-B1-6A-1_2.fq.gz;NAM-B1-6D-1_1.fq.gz;NAM-B1-6D-1_2.fq.gz;NF-YB-1B-1_1.fq.gz;NF-YB-1B-1_2.fq.gz;PIF-5B-1_1.fq.gz;PIF-5B-1_2.fq.gz;PIF-5D-1_1.fq.gz;PIF-5D-1_2.fq.gz;PLATZ-A_1.fq.gz;PLATZ-A_2.fq.gz;PLATZ-B_1.fq.gz;PLATZ-B_2.fq.gz;PLATZ-D_1.fq.gz;PLATZ-D_2.fq.gz;Q-5A-1_1.fq.gz;Q-5A-1_2.fq.gz;Q-5B-1_1.fq.gz;Q-5B-1_2.fq.gz;REF6-3D-1_ampDAP_1.fq.gz;REF6-3D-1_ampDAP_2.fq.gz;REF6-3D-1_1.fq.gz;REF6-3D-1_2.fq.gz;Rht-4B-1_1.fq.gz;Rht-4B-1_2.fq.gz;Rht-4D-1_1.fq.gz;Rht-4D-1_2.fq.gz;RWP-RK-2D-1_1.fq.gz;RWP-RK-2D-1_2.fq.gz;SBP-7B-2_1.fq.gz;SBP-7B-2_2.fq.gz;SPL16-gw8-7B-1_1.fq.gz;SPL16-gw8-7B-1_2.fq.gz;SPL16-gw8-7D-1_1.fq.gz;SPL16-gw8-7D-1_2.fq.gz;Tify-5A-1_1.fq.gz;Tify-5A-1_2.fq.gz;Tify-5B-1_1.fq.gz;Tify-5B-1_2.fq.gz;Tify-6B-1_1.fq.gz;Tify-6B-1_2.fq.gz;Trihelix-2A-1_1.fq.gz;Trihelix-2A-1_2.fq.gz;Trihelix-2B-1_1.fq.gz;Trihelix-2B-1_2.fq.gz;TUB-1B-1_1.fq.gz;TUB-1B-1_2.fq.gz;TUB-3A-1_1.fq.gz;TUB-3A-1_2.fq.gz;Vrn1-5D-1_1.fq.gz;Vrn1-5D-1_2.fq.gz;WRKY-1D-1_1.fq.gz;WRKY-1D-1_2.fq.gz;WRKY-1D-2_1.fq.gz;WRKY-1D-2_2.fq.gz;WRKY-6A-1_1.fq.gz;WRKY-6A-1_2.fq.gz;Halo-1_1.fq.gz;Halo-1_2.fq.gz;Halo-2_1.fq.gz;Halo-2_2.fq.gz;Halo-3_1.fq.gz;Halo-3_2.fq.gz;20220211_CS-GNI-2D_1.fq.gz;20220211_CS-GNI-2D_2.fq.gz;20220211_CS-GNI-U_1.fq.gz;20220211_CS-GNI-U_2.fq.gz;20220211_CS-TaMOR-4B_1.fq.gz;20220211_CS-TaMOR-4B_2.fq.gz;20220211_CS-TaMOR-4D_1.fq.gz;20220211_CS-TaMOR-4D_2.fq.gz;20220211_CS-Tamyb10-3B_1.fq.gz;20220211_CS-Tamyb10-3B_2.fq.gz;20220211_CS-Tamyb10-3D_1.fq.gz;20220211_CS-Tamyb10-3D_2.fq.gz;20220211_CS-TaSEP3-7A_1.fq.gz;20220211_CS-TaSEP3-7A_2.fq.gz;20220211_CS-TaSEP3-7B_1.fq.gz;20220211_CS-TaSEP3-7B_2.fq.gz;20220211_CS-TaSEP3-7D_1.fq.gz;20220211_CS-TaSEP3-7D_2.fq.gz;20220211_CS-TaVRT2-7D_1.fq.gz;20220211_CS-TaVRT2-7D_2.fq.gz;20220211_CS-TdDof-3A_1.fq.gz;20220211_CS-TdDof-3A_2.fq.gz

Peak files: Alfin-4D-1_PE_peaks.filter.p10.bed;AP2-B_PE_peaks.filter.p10.bed;AP2-D_PE_peaks.filter.p10.bed;AP2-DREB-1A-1_PE_peaks.filter.p10.bed;AP2-DREB-1B-1_PE_peaks.filter.p10.bed;AP2-DREB-1D-1_PE_peaks.filter.p10.bed;AP2-

DREB-2D-1_PE_peaks.filter.p10.bed;AP2-DREB-5B-3_PE_peaks.filter.p10.bed;AP2-ERF-3A-1_PE_peaks.filter.p10.bed;AP2-ERF-4B-2_PE_peaks.filter.p10.bed;AP2-ERF-5B-1_PE_peaks.filter.p10.bed;AP2-ERF-5B-2_PE_peaks.filter.p10.bed;AP2-ERF-5D-1_PE_peaks.filter.p10.bed;AP2-ERF-6A-1_PE_peaks.filter.p10.bed;AP2-ERF-6A-2_PE_peaks.filter.p10.bed;AP2-ERF-6D-1_PE_peaks.filter.p10.bed;AP2-RAV-4B-1_PE_peaks.filter.p10.bed;B3-2A-1_PE_peaks.filter.p10.bed;B3-2B-2_PE_peaks.filter.p10.bed;B3-4A-1_PE_peaks.filter.p10.bed;B3-4B-1_PE_peaks.filter.p10.bed;B3-4B-2_PE_peaks.filter.p10.bed;B3-5A-1_PE_peaks.filter.p10.bed;B3-5B-1_PE_peaks.filter.p10.bed;B3-5D-2_PE_peaks.filter.p10.bed;B3-6D-1_PE_peaks.filter.p10.bed;B3-6D-2_PE_peaks.filter.p10.bed;B3-7B-1_PE_peaks.filter.p10.bed;B3-7D-1_PE_peaks.filter.p10.bed;B3-ARF-2D-1_PE_peaks.filter.p10.bed;B3-ARF-3A-1_PE_peaks.filter.p10.bed;B3-ARF-3B-2_PE_peaks.filter.p10.bed;BES1-6A-1_PE_peaks.filter.p10.bed;BES1-A_PE_peaks.filter.p10.bed;BES1-B_PE_peaks.filter.p10.bed;BES1-D_PE_peaks.filter.p10.bed;bHLH-1A-1_PE_peaks.filter.p10.bed;bHLH-3A-2_PE_peaks.filter.p10.bed;bHLH-4B-1_PE_peaks.filter.p10.bed;bHLH-5A-1_PE_peaks.filter.p10.bed;bHLH-5A-4_PE_peaks.filter.p10.bed;bHLH-6A-1_PE_peaks.filter.p10.bed;bHLH-7A-2_PE_peaks.filter.p10.bed;bHLH-7A-3_PE_peaks.filter.p10.bed;bHLH-7D-2_PE_peaks.filter.p10.bed;bHLH-U_PE_peaks.filter.p10.bed;bZIP-2A-2_PE_peaks.filter.p10.bed;bZIP-2A-3_PE_peaks.filter.p10.bed;bZIP-3A-1_PE_peaks.filter.p10.bed;bZIP-3A-2_PE_peaks.filter.p10.bed;bZIP-5A-1_PE_peaks.filter.p10.bed;bZIP-5B-3_PE_peaks.filter.p10.bed;bZIP-6A-1_PE_peaks.filter.p10.bed;bZIP-7A-1_PE_peaks.filter.p10.bed;bZIP-A_PE_peaks.filter.p10.bed;bZIP-B_PE_peaks.filter.p10.bed;bZIP-D_PE_peaks.filter.p10.bed;C2-A_PE_peaks.filter.p10.bed;C2-B_PE_peaks.filter.p10.bed;C2C2-Dof-2A-1_PE_peaks.filter.p10.bed;C2C2-Dof-2D-1_PE_peaks.filter.p10.bed;C2C2-Dof-4A-1_PE_peaks.filter.p10.bed;C2C2-Dof-4D-1_PE_peaks.filter.p10.bed;C2-D_PE_peaks.filter.p10.bed;C2H2-2A-1_PE_peaks.filter.p10.bed;C2H2-3A-1_PE_peaks.filter.p10.bed;C2H2-3D-2_PE_peaks.filter.p10.bed;C2H2-4A-1_PE_peaks.filter.p10.bed;C2H2-5B-1_PE_peaks.filter.p10.bed;C2H2-6A-1_PE_peaks.filter.p10.bed;C2H2-6B-1_PE_peaks.filter.p10.bed;C2H2-6D-2_PE_peaks.filter.p10.bed;C3H-1A-1_PE_peaks.filter.p10.bed;C3H-1B-1_PE_peaks.filter.p10.bed;C3H-1D-2_PE_peaks.filter.p10.bed;C3H-3A-1_PE_peaks.filter.p10.bed;C3H-3A-2_PE_peaks.filter.p10.bed;C3H-4B-1_PE_peaks.filter.p10.bed;C3H-7B-1_PE_peaks.filter.p10.bed;DBB-7A-1_PE_peaks.filter.p10.bed;DBP-1B-1_PE_peaks.filter.p10.bed;DBP-1D-1_PE_peaks.filter.p10.bed;EIL-4D-1_PE_peaks.filter.p10.bed;GARP-ARR-6A-1_PE_peaks.filter.p10.bed;GARP-ARR-7A-1_PE_peaks.filter.p10.bed;GARP-ARR-7A-3_PE_peaks.filter.p10.bed;GARP-G2-2A-1_PE_peaks.filter.p10.bed;GARP-G2-2B-1_PE_peaks.filter.p10.bed;GARP-G2-3B-1_PE_peaks.filter.p10.bed;GARP-G2-4A-1_PE_peaks.filter.p10.bed;GARP-G2-4D-2_PE_peaks.filter.p10.bed;GARP-G2-5B-3_PE_peaks.filter.p10.bed;GRAS-2A-1_PE_peaks.filter.p10.bed;GRAS-4B-1_PE_peaks.filter.p10.bed;GRAS-4B-2_PE_peaks.filter.p10.bed;GRF4-2A-1_PE_peaks.filter.p10.bed;GRF4-2B-1_PE_peaks.filter.p10.bed;GRF4-2D-1_PE_peaks.filter.p10.bed;GRF-6A-1_PE_peaks.filter.p10.bed;HB-BELL-4B-1_PE_peaks.filter.p10.bed;HB-BELL-5D-1_PE_peaks.filter.p10.bed;HB-HD-ZIP-2A-1_PE_peaks.filter.p10.bed;HB-HD-ZIP-2A-2_PE_peaks.filter.p10.bed;HB-HD-ZIP-2B-1_PE_peaks.filter.p10.bed;HB-HD-ZIP-6A-1_PE_peaks.filter.p10.bed;HB-HD-ZIP-6A-2_PE_peaks.filter.p10.bed;HB-HD-ZIP-6B-2_PE_peaks.filter.p10.bed;HB-PHD-6A-1_PE_peaks.filter.p10.bed;HB-WOX-A_PE_peaks.filter.p10.bed;HB-WOX-B_PE_peaks.filter.p10.bed;HB-WOX-D_PE_peaks.filter.p10.bed;HSF-U_PE_peaks.filter.p10.bed;IPA1-7B-1_PE_peaks.filter.p10.bed;LIM-7B-1_PE_peaks.filter.p10.bed;LIM-7D-1_PE_peaks.filter.p10.bed;LOB-1A-1_PE_peaks.filter.p10.bed;LOB-3A-1_PE_peaks.filter.p10.bed;LOB-3B-1_PE_peaks.filter.p10.bed;LOB-3D-1_PE_peaks.filter.p10.bed;LOB-3D-2_PE_peaks.filter.p10.bed;MADS1-lgy3-4A-1_PE_peaks.filter.p10.bed;MADS1-lgy3-4B-1_PE_peaks.filter.p10.bed;MADS1-lgy3-4D-1_PE_peaks.filter.p10.bed;MADS-M-4D-1_PE_peaks.filter.p10.bed;MADS-MIKC-5A-1_PE_peaks.filter.p10.bed;MADS-MIKC-7A-1_PE_peaks.filter.p10.bed;MYB-1A-1_PE_peaks.filter.p10.bed;MYB-1A-2_PE_peaks.filter.p10.bed;MYB-2A-1_PE_peaks.filter.p10.bed;MYB-2A-2_PE_peaks.filter.p10.bed;MYB-3A-1_PE_peaks.filter.p10.bed;MYB-3D-1_PE_peaks.filter.p10.bed;MYB-4A-1_PE_peaks.filter.p10.bed;MYB-4A-2_PE_peaks.filter.p10.bed;MYB-5A-1_PE_peaks.filter.p10.bed;MYB-5B-1_PE_peaks.filter.p10.bed;MYB-6D-1_PE_peaks.filter.p10.bed;MYB-7A-3_PE_peaks.filter.p10.bed;MYB-7A-5_PE_peaks.filter.p10.bed;NAC-1D-1_PE_peaks.filter.p10.bed;NAC-2A-1_PE_peaks.filter.p10.bed;NAC-2A-3_PE_peaks.filter.p10.bed;NAC-2A-4_PE_peaks.filter.p10.bed;NAC-2D-6_PE_peaks.filter.p10.bed;NAC-3A-1_PE_peaks.filter.p10.bed;NAC-3D-1_PE_peaks.filter.p10.bed;NAC-6A-1_PE_peaks.filter.p10.bed;NAC-6B-1_PE_peaks.filter.p10.bed;NAC-6D-1_PE_peaks.filter.p10.bed;NAC-7A-1_PE_peaks.filter.p10.bed;NAC-7A-2_PE_peaks.filter.p10.bed;NAC-7B-2_PE_peaks.filter.p10.bed;NAC-7D-2_PE_peaks.filter.p10.bed;NAM-B1-6A-1_PE_peaks.filter.p10.bed;NAM-B1-6D-1_PE_peaks.filter.p10.bed;NF-YB-1B-1_PE_peaks.filter.p10.bed;PIF-5B-1_PE_peaks.filter.p10.bed;PIF-5D-1_PE_peaks.filter.p10.bed;PLATZ-A_PE_peaks.filter.p10.bed;PLATZ-B_PE_peaks.filter.p10.bed;PLATZ-D_PE_peaks.filter.p10.bed;Q-5A-1_PE_peaks.filter.p10.bed;Q-5B-1_PE_peaks.filter.p10.bed;REF6-3D-1_ampDAP_PE_peaks.filter.p10.bed;REF6-3D-1_PE_peaks.filter.p10.bed;Rht-4B-1_PE_peaks.filter.p10.bed;Rht-4D-1_PE_peaks.filter.p10.bed;RWP-RK-2D-1_PE_peaks.filter.p10.bed;SBP-7B-2_PE_peaks.filter.p10.bed;SPL16-gw8-7B-1_PE_peaks.filter.p10.bed;SPL16-gw8-7D-1_PE_peaks.filter.p10.bed;Tify-5A-1_PE_peaks.filter.p10.bed;Tify-5B-1_PE_peaks.filter.p10.bed;Tify-6B-1_PE_peaks.filter.p10.bed;Trihelix-2A-1_PE_peaks.filter.p10.bed;Trihelix-2B-1_PE_peaks.filter.p10.bed;TUB-1B-1_PE_peaks.filter.p10.bed;TUB-3A-1_PE_peaks.filter.p10.bed;Vrn1-5D-1_PE_peaks.filter.p10.bed;WRKY-1D-1_PE_peaks.filter.p10.bed;WRKY-1D-2_PE_peaks.filter.p10.bed;WRKY-6A-1_PE_peaks.filter.p10.bed;Halo-merge_PE_peaks.bed;GNI-2D_PE_peaks.filter.p10.bed;GNI-U_PE_peaks.filter.p10.bed;TaMOR-4B_PE_peaks.filter.p10.bed;TaMOR-4D_PE_peaks.filter.p10.bed;Tamyb10-3B_PE_peaks.filter.p10.bed;Tamyb10-3D_PE_peaks.filter.p10.bed;TaSEP3-7A_PE_peaks.filter.p10.bed;TaSEP3-7B_PE_peaks.filter.p10.bed;TaSEP3-7D_PE_peaks.filter.p10.bed;TaVRT2-7D_PE_peaks.filter.p10.bed;TdDof-3A_PE_peaks.filter.p10.bed

bw files: Alfin-4D-1.sort.q20.rmdup.rpm.bw;AP2-B.sort.q20.rmdup.rpm.bw;AP2-D.sort.q20.rmdup.rpm.bw;AP2-DREB-1A-1.sort.q20.rmdup.rpm.bw;AP2-DREB-1B-1.sort.q20.rmdup.rpm.bw;AP2-DREB-1D-1.sort.q20.rmdup.rpm.bw;AP2-DREB-2D-1.sort.q20.rmdup.rpm.bw;AP2-DREB-5B-3.sort.q20.rmdup.rpm.bw;AP2-ERF-3A-1.sort.q20.rmdup.rpm.bw;AP2-ERF-4B-2.sort.q20.rmdup.rpm.bw;AP2-ERF-5B-1.sort.q20.rmdup.rpm.bw;AP2-ERF-5B-2.sort.q20.rmdup.rpm.bw;AP2-ERF-5D-1.sort.q20.rmdup.rpm.bw;AP2-ERF-6A-1.sort.q20.rmdup.rpm.bw;AP2-ERF-6A-2.sort.q20.rmdup.rpm.bw;AP2-ERF-6D-1.sort.q20.rmdup.rpm.bw;AP2-RAV-4B-1.sort.q20.rmdup.rpm.bw;B3-2A-1.sort.q20.rmdup.rpm.bw;B3-2B-2.sort.q20.rmdup.rpm.bw;B3-4A-1.sort.q20.rmdup.rpm.bw;B3-4B-1.sort.q20.rmdup.rpm.bw;B3-4B-2.sort.q20.rmdup.rpm.bw;B3-5A-1.sort.q20.rmdup.rpm.bw;B3-5B-1.sort.q20.rmdup.rpm.bw;B3-5D-2.sort.q20.rmdup.rpm.bw;B3-6D-1.sort.q20.rmdup.rpm.bw;B3-6D-2.sort.q20.rmdup.rpm.bw;B3-7B-1.sort.q20.rmdup.rpm.bw;B3-7D-1.sort.q20.rmdup.rpm.bw;B3-ARF-2D-1.sort.q20.rmdup.rpm.bw;B3-ARF-3A-1.sort.q20.rmdup.rpm.bw;B3-ARF-3B-2.sort.q20.rmdup.rpm.bw;BES1-6A-1.sort.q20.rmdup.rpm.bw;BES1-A.sort.q20.rmdup.rpm.bw;BES1-B.sort.q20.rmdup.rpm.bw;BES1-D.sort.q20.rmdup.rpm.bw;bHLH-1A-1.sort.q20.rmdup.rpm.bw;bHLH-3A-2.sort.q20.rmdup.rpm.bw;bHLH-4B-1.sort.q20.rmdu

p.rpm.bw;bHLH-5A-1.sort.q20.rmdup.rpm.bw;bHLH-5A-4.sort.q20.rmdup.rpm.bw;bHLH-6A-1.sort.q20.rmdup.rpm.bw;bHLH-7A-2.sort.q20.rmdup.rpm.bw;bHLH-7A-3.sort.q20.rmdup.rpm.bw;bHLH-7D-2.sort.q20.rmdup.rpm.bw;bHLH-U.sort.q20.rmdup.rpm.bw;bZIP-2A-2.sort.q20.rmdup.rpm.bw;bZIP-2A-3.sort.q20.rmdup.rpm.bw;bZIP-3A-1.sort.q20.rmdup.rpm.bw;bZIP-3A-2.sort.q20.rmdup.rpm.bw;bZIP-5A-1.sort.q20.rmdup.rpm.bw;bZIP-5B-3.sort.q20.rmdup.rpm.bw;bZIP-6A-1.sort.q20.rmdup.rpm.bw;bZIP-7A-1.sort.q20.rmdup.rpm.bw;bZIP-A.sort.q20.rmdup.rpm.bw;bZIP-B.sort.q20.rmdup.rpm.bw;bZIP-D.sort.q20.rmdup.rpm.bw;C2-A.sort.q20.rmdup.rpm.bw;C2-B.sort.q20.rmdup.rpm.bw;C2C2-Dof-2A-1.sort.q20.rmdup.rpm.bw;C2C2-Dof-2D-1.sort.q20.rmdup.rpm.bw;C2C2-Dof-4A-1.sort.q20.rmdup.rpm.bw;C2C2-Dof-4D-1.sort.q20.rmdup.rpm.bw;C2-D.sort.q20.rmdup.rpm.bw;C2H2-2A-1.sort.q20.rmdup.rpm.bw;C2H2-3A-1.sort.q20.rmdup.rpm.bw;C2H2-3D-2.sort.q20.rmdup.rpm.bw;C2H2-4A-1.sort.q20.rmdup.rpm.bw;C2H2-5B-1.sort.q20.rmdup.rpm.bw;C2H2-6A-1.sort.q20.rmdup.rpm.bw;C2H2-6B-1.sort.q20.rmdup.rpm.bw;C2H2-6D-2.sort.q20.rmdup.rpm.bw;C3H-1A-1.sort.q20.rmdup.rpm.bw;C3H-1B-1.sort.q20.rmdup.rpm.bw;C3H-1D-2.sort.q20.rmdup.rpm.bw;C3H-3A-1.sort.q20.rmdup.rpm.bw;C3H-3A-2.sort.q20.rmdup.rpm.bw;C3H-4B-1.sort.q20.rmdup.rpm.bw;C3H-7B-1.sort.q20.rmdup.rpm.bw;DBB-7A-1.sort.q20.rmdup.rpm.bw;DBP-1B-1.sort.q20.rmdup.rpm.bw;DBP-1D-1.sort.q20.rmdup.rpm.bw;EIL-4D-1.sort.q20.rmdup.rpm.bw;GARP-ARR-6A-1.sort.q20.rmdup.rpm.bw;GARP-ARR-7A-1.sort.q20.rmdup.rpm.bw;GARP-ARR-7A-3.sort.q20.rmdup.rpm.bw;GARP-G2-2A-1.sort.q20.rmdup.rpm.bw;GARP-G2-2B-1.sort.q20.rmdup.rpm.bw;GARP-G2-3B-1.sort.q20.rmdup.rpm.bw;GARP-G2-4A-1.sort.q20.rmdup.rpm.bw;GARP-G2-4D-2.sort.q20.rmdup.rpm.bw;GARP-G2-5B-3.sort.q20.rmdup.rpm.bw;GRAS-2A-1.sort.q20.rmdup.rpm.bw;GRAS-4B-1.sort.q20.rmdup.rpm.bw;GRAS-4B-2.sort.q20.rmdup.rpm.bw;GRF4-2A-1.sort.q20.rmdup.rpm.bw;GRF4-2B-1.sort.q20.rmdup.rpm.bw;GRF4-2D-1.sort.q20.rmdup.rpm.bw;GRF-6A-1.sort.q20.rmdup.rpm.bw;Halo-merge.rpm.bw;HB-BELL-4B-1.sort.q20.rmdup.rpm.bw;HB-BELL-5D-1.sort.q20.rmdup.rpm.bw;HB-HD-ZIP-2A-1.sort.q20.rmdup.rpm.bw;HB-HD-ZIP-2A-2.sort.q20.rmdup.rpm.bw;HB-HD-ZIP-2B-1.sort.q20.rmdup.rpm.bw;HB-HD-ZIP-6A-1.sort.q20.rmdup.rpm.bw;HB-HD-ZIP-6A-2.sort.q20.rmdup.rpm.bw;HB-HD-ZIP-6B-2.sort.q20.rmdup.rpm.bw;HB-PHD-6A-1.sort.q20.rmdup.rpm.bw;HB-WOX-A.sort.q20.rmdup.rpm.bw;HB-WOX-B.sort.q20.rmdup.rpm.bw;HB-WOX-D.sort.q20.rmdup.rpm.bw;HSF-U.sort.q20.rmdup.rpm.bw;IPA1-7B-1.sort.q20.rmdup.rpm.bw;LIM-7B-1.sort.q20.rmdup.rpm.bw;LIM-7D-1.sort.q20.rmdup.rpm.bw;LOB-1A-1.sort.q20.rmdup.rpm.bw;LOB-3A-1.sort.q20.rmdup.rpm.bw;LOB-3B-1.sort.q20.rmdup.rpm.bw;LOB-3D-1.sort.q20.rmdup.rpm.bw;LOB-3D-2.sort.q20.rmdup.rpm.bw;MADS1-lgy3-4A-1.sort.q20.rmdup.rpm.bw;MADS1-lgy3-4B-1.sort.q20.rmdup.rpm.bw;MADS1-lgy3-4D-1.sort.q20.rmdup.rpm.bw;MADS-M-4D-1.sort.q20.rmdup.rpm.bw;MADS-MIKC-5A-1.sort.q20.rmdup.rpm.bw;MADS-MIKC-7A-1.sort.q20.rmdup.rpm.bw;MYB-1A-1.sort.q20.rmdup.rpm.bw;MYB-1A-2.sort.q20.rmdup.rpm.bw;MYB-2A-1.sort.q20.rmdup.rpm.bw;MYB-2A-2.sort.q20.rmdup.rpm.bw;MYB-3A-1.sort.q20.rmdup.rpm.bw;MYB-3D-1.sort.q20.rmdup.rpm.bw;MYB-4A-1.sort.q20.rmdup.rpm.bw;MYB-4A-2.sort.q20.rmdup.rpm.bw;MYB-5A-1.sort.q20.rmdup.rpm.bw;MYB-5B-1.sort.q20.rmdup.rpm.bw;MYB-6D-1.sort.q20.rmdup.rpm.bw;MYB-7A-3.sort.q20.rmdup.rpm.bw;MYB-7A-5.sort.q20.rmdup.rpm.bw;NAC-1D-1.sort.q20.rmdup.rpm.bw;NAC-2A-1.sort.q20.rmdup.rpm.bw;NAC-2A-3.sort.q20.rmdup.rpm.bw;NAC-2A-4.sort.q20.rmdup.rpm.bw;NAC-2D-6.sort.q20.rmdup.rpm.bw;NAC-3A-1.sort.q20.rmdup.rpm.bw;NAC-3D-1.sort.q20.rmdup.rpm.bw;NAC-6A-1.sort.q20.rmdup.rpm.bw;NAC-6B-1.sort.q20.rmdup.rpm.bw;NAC-6D-1.sort.q20.rmdup.rpm.bw;NAC-7A-1.sort.q20.rmdup.rpm.bw;NAC-7A-2.sort.q20.rmdup.rpm.bw;NAC-7B-2.sort.q20.rmdup.rpm.bw;NAC-7D-2.sort.q20.rmdup.rpm.bw;NAM-B1-6A-1.sort.q20.rmdup.rpm.bw;NAM-B1-6D-1.sort.q20.rmdup.rpm.bw;NF-YB-1B-1.sort.q20.rmdup.rpm.bw;PIF-5B-1.sort.q20.rmdup.rpm.bw;PIF-5D-1.sort.q20.rmdup.rpm.bw;PLATZ-A.sort.q20.rmdup.rpm.bw;PLATZ-B.sort.q20.rmdup.rpm.bw;PLATZ-D.sort.q20.rmdup.rpm.bw;Q-5A-1.sort.q20.rmdup.rpm.bw;Q-5B-1.sort.q20.rmdup.rpm.bw;REF6-3D-1_ampDAP.sort.q20.rmdup.rpm.bw;REF6-3D-1.sort.q20.rmdup.rpm.bw;Rht-4B-1.sort.q20.rmdup.rpm.bw;Rht-4D-1.sort.q20.rmdup.rpm.bw;RWP-RK-2D-1.sort.q20.rmdup.rpm.bw;SBP-7B-2.sort.q20.rmdup.rpm.bw;SPL16-gw8-7B-1.sort.q20.rmdup.rpm.bw;SPL16-gw8-7D-1.sort.q20.rmdup.rpm.bw;Tify-5A-1.sort.q20.rmdup.rpm.bw;Tify-5B-1.sort.q20.rmdup.rpm.bw;Tify-6B-1.sort.q20.rmdup.rpm.bw;Trihelix-2A-1.sort.q20.rmdup.rpm.bw;Trihelix-2B-1.sort.q20.rmdup.rpm.bw;TUB-1B-1.sort.q20.rmdup.rpm.bw;TUB-3A-1.sort.q20.rmdup.rpm.bw;Vrn1-5D-1.sort.q20.rmdup.rpm.bw;WRKY-1D-1.sort.q20.rmdup.rpm.bw;WRKY-1D-2.sort.q20.rmdup.rpm.bw;WRKY-6A-1.sort.q20.rmdup.rpm.bw;GNI-2D.sort.q20.rmdup.rpm.bw;GNI-U.sort.q20.rmdup.rpm.bw;TaMOR-4B.sort.q20.rmdup.rpm.bw;TaMOR-4D.sort.q20.rmdup.rpm.bw;Tamyb10-3B.sort.q20.rmdup.rpm.bw;Tamyb10-3D.sort.q20.rmdup.rpm.bw;TaSEP3-7A.sort.q20.rmdup.rpm.bw;TaSEP3-7B.sort.q20.rmdup.rpm.bw;TaSEP3-7D.sort.q20.rmdup.rpm.bw;TaVRT2-7D.sort.q20.rmdup.rpm.bw;TdDof-3A.sort.q20.rmdup.rpm.bw

Genome browser session
(e.g. UCSC)

http://bioinfo.sibs.ac.cn/dap-seq_CS_jbrowse/

Methodology

Replicates

AP2-DREB-1A-1, AP2-DREB-1B-1, AP2-DREB-1D-1, AP2-ERF-6A-1 with two biological replicates (Fig. S9 and Fig. S13).

Sequencing depth

All DAP-seq reads were paired-end 150bp. The number of raw reads and mapped reads (q > 20 and PCR duplicates removed) were show below:

TF raw_reads mapped_reads
Alfin-4D-1 55,009,100 19,689,151
AP2-B 70,829,430 12,971,559
AP2-D 118,943,322 30,049,193
AP2-DREB-1A-1 123,122,794 46,388,339
AP2-DREB-1B-1 160,151,212 51,840,155
AP2-DREB-1D-1 166,769,898 53,758,258
AP2-DREB-2D-1 138,967,776 71,018,771
AP2-DREB-5B-3 62,945,094 26,997,903
AP2-ERF-3A-1 155,541,720 10,279,445
AP2-ERF-4B-2 90,995,392 22,182,956
AP2-ERF-5B-1 51,626,096 27,662,222
AP2-ERF-5B-2 80,049,018 38,960,090
AP2-ERF-5D-1 21,859,116 9,165,569

AP2-ERF-6A-1 90,533,246 15,683,919
AP2-ERF-6A-2 44,479,564 15,478,423
AP2-ERF-6D-1 91,120,804 13,797,802
AP2-RAV-4B-1 85,839,768 54,655,218
B3-2A-1 95,680,130 13,468,080
B3-2B-2 43,393,962 9,389,401
B3-4A-1 77,311,956 10,827,336
B3-4B-1 65,755,304 10,900,078
B3-4B-2 21,319,906 2,885,777
B3-5A-1 60,256,972 7,084,077
B3-5B-1 68,370,206 9,036,174
B3-5D-2 36,026,250 5,348,336
B3-6D-1 12,722,186 2,257,540
B3-6D-2 77,502,176 9,453,211
B3-7B-1 96,640,536 13,620,553
B3-7D-1 109,964,842 14,851,470
B3-ARF-2D-1 41,541,530 13,175,965
B3-ARF-3A-1 81,368,118 11,958,287
B3-ARF-3B-2 41,796,286 11,168,249
BES1-6A-1 55,760,022 19,940,467
BES1-A 63,161,308 24,112,754
BES1-B 77,264,522 27,482,981
BES1-D 67,965,750 23,834,253
bHLH-1A-1 37,524,668 18,053,634
bHLH-3A-2 54,346,954 5,839,845
bHLH-4B-1 42,696,912 16,996,941
bHLH-5A-1 50,441,536 8,378,814
bHLH-5A-4 21,555,530 3,668,223
bHLH-6A-1 44,877,492 7,169,096
bHLH-7A-2 21,468,076 5,544,651
bHLH-7A-3 29,859,530 6,703,328
bHLH-7D-2 31,487,904 8,866,724
bHLH-U 39,570,572 6,730,247
bZIP-2A-2 38,644,394 13,411,524
bZIP-2A-3 51,931,212 22,177,905
bZIP-3A-1 90,971,558 55,697,499
bZIP-3A-2 163,403,766 104,336,039
bZIP-5A-1 61,359,848 17,524,656
bZIP-5B-3 110,357,470 64,110,141
bZIP-6A-1 26,633,698 6,747,306
bZIP-7A-1 65,040,060 42,425,714
bZIP-A 86,922,542 14,963,020
bZIP-B 117,318,182 16,121,077
bZIP-D 81,336,132 11,306,481
C2-A 12,479,536 4,808,854
C2-B 6,790,040 2,578,201
C2C2-Dof-2A-1 80,659,996 14,024,563
C2C2-Dof-2D-1 69,815,316 11,920,330
C2C2-Dof-4A-1 78,514,658 35,468,366
C2C2-Dof-4D-1 86,827,478 35,211,048
C2-D 9,423,056 3,971,008
C2H2-2A-1 137,936,616 60,281,599
C2H2-3A-1 107,733,744 37,262,136
C2H2-3D-2 51,222,352 17,471,085
C2H2-4A-1 43,410,668 18,371,682
C2H2-5B-1 17,069,050 3,415,589
C2H2-6A-1 42,264,124 9,713,026
C2H2-6B-1 20,833,098 7,004,265
C2H2-6D-2 82,709,502 8,233,337
C3H-1A-1 53,548,242 8,413,518
C3H-1D-2 37,601,504 7,673,397
C3H-3A-1 30,715,470 15,291,443
C3H-3A-2 55,463,244 15,484,599
C3H-7B-1 22,160,948 4,521,051
DBB-7A-1 35,884,136 4,550,475
DBP-1B-1 50,864,722 6,548,695
DBP-1D-1 43,270,114 5,657,622
EIL-4D-1 52,580,906 11,861,565

GARP-ARR-6A-1 72,836,418 16,861,457
GARP-ARR-7A-1 46,385,926 5,522,207
GARP-ARR-7A-3 49,942,646 6,022,140
GARP-G2-2A-1 128,563,374 66,079,363
GARP-G2-2B-1 116,315,468 32,402,132
GARP-G2-3B-1 50,751,050 7,958,844
GARP-G2-4A-1 96,368,650 22,757,854
GARP-G2-4D-2 70,031,588 37,244,870
GARP-G2-5B-3 160,127,610 39,608,807
GNI-2D 45,999,758 22,514,143
GNI-U 53,919,062 22,541,915
GRAS-2A-1 21,998,140 9,883,083
GRAS-4B-2 65,429,870 6,485,100
GRF4-2A-1 52,860,800 14,419,128
GRF4-2B-1 63,830,928 19,809,060
GRF4-2D-1 44,699,428 16,632,855
GRF-6A-1 92,825,442 16,052,200
HB-BELL-4B-1 71,353,844 14,351,300
HB-BELL-5D-1 67,235,040 10,779,044
HB-HD-ZIP-2A-1 24,586,010 12,158,542
HB-HD-ZIP-2A-2 59,190,696 23,294,154
HB-HD-ZIP-2B-1 35,132,180 9,122,619
HB-HD-ZIP-6A-1 53,793,388 31,374,482
HB-HD-ZIP-6A-2 60,728,028 14,986,024
HB-HD-ZIP-6B-2 59,949,072 15,204,030
HB-PHD-6A-1 86,870,186 31,686,694
HB-WOX-A 3,134,048 1,309,940
HB-WOX-B 4,612,136 1,566,386
HB-WOX-D 2,833,386 1,263,046
HSF-U 30,208,124 12,793,545
IPA1-7B-1 57,540,476 19,639,449
LIM-7B-1 59,620,982 23,772,393
LIM-7D-1 62,426,170 19,506,992
LOB-1A-1 25,877,258 10,246,768
LOB-3A-1 40,391,124 8,911,961
LOB-3B-1 26,984,848 7,108,793
LOB-3D-1 45,258,494 9,454,122
LOB-3D-2 47,184,314 8,515,444
MADS1-lgy3-4A-1 92,699,698 21,721,010
MADS1-lgy3-4B-1 23,524,664 7,636,929
MADS1-lgy3-4D-1 46,510,940 11,539,835
MADS-M-4D-1 64,031,630 7,620,777
MADS-MIKC-5A-1 49,216,104 23,183,259
MADS-MIKC-7A-1 59,255,066 7,608,336
MYB-1A-1 55,393,490 5,703,732
MYB-1A-2 75,142,620 7,855,907
MYB-2A-1 74,602,568 8,979,639
MYB-2A-2 87,494,586 10,255,650
MYB-3A-1 26,598,050 12,676,840
MYB-3D-1 58,027,346 7,771,393
MYB-4A-1 77,285,006 7,925,038
MYB-4A-2 60,080,772 5,767,530
MYB-5A-1 154,255,784 15,407,933
MYB-5B-1 90,658,504 8,200,771
MYB-6D-1 162,781,464 49,461,208
MYB-7A-3 100,118,238 63,345,793
MYB-7A-5 40,704,660 7,604,570
NAC-1D-1 37,179,260 4,944,215
NAC-2A-1 48,705,844 23,474,274
NAC-2A-3 110,230,986 17,308,375
NAC-2A-4 146,410,732 18,429,403
NAC-2D-6 49,905,060 6,235,623
NAC-3A-1 169,423,432 56,466,856
NAC-3D-1 126,989,858 30,368,126
NAC-6A-1 146,620,102 42,829,632
NAC-6B-1 180,218,562 42,379,353
NAC-6D-1 120,707,436 29,103,419
NAC-7A-1 76,424,528 8,302,185

NAC-7A-2 52,247,296 11,168,524
NAC-7B-2 68,506,578 11,746,421
NAC-7D-2 87,454,148 15,556,111
NAM-B1-6A-1 74,131,190 34,515,759
NAM-B1-6D-1 66,949,012 39,152,598
NF-YB-1B-1 28,823,556 3,902,526
PIF-5B-1 65,754,164 24,982,431
PIF-5D-1 68,345,658 24,625,944
PLATZ-A 76,759,158 22,794,684
PLATZ-B 85,546,406 22,901,495
PLATZ-D 71,006,516 20,923,779
Q-5A-1 70,132,144 27,405,111
Q-5B-1 50,809,016 5,667,335
REF6-3D-1 108,122,454 40,632,219
REF6-3D-1_ampDAP 68,236,362 7,794,211
Rht-4B-1 73,947,030 11,707,690
Rht-4D-1 86,263,286 10,856,621
RWP-RK-2D-1 68,020,216 9,612,135
SBP-7B-2 92,861,456 11,896,682
SPL16-gw8-7B-1 85,198,998 37,154,176
SPL16-gw8-7D-1 89,686,500 25,387,552
TaMOR-4B 54,948,490 23,542,528
TaMOR-4D 41,410,958 18,297,595
Tamyb10-3B 38,604,326 18,996,085
Tamyb10-3D 36,497,072 17,264,955
TaSEP3-7A 106,775,486 35,649,127
TaSEP3-7B 16,886 2,756
TaSEP3-7D 2,436 185
TaVRT2-7D 43,741,480 18,882,545
TdDof-3A 45,935,788 20,778,765
Tify-5A-1 55,446,618 26,460,902
Tify-5B-1 67,757,274 16,629,259
Tify-6B-1 66,982,266 15,111,212
Trihelix-2A-1 79,676,848 23,483,438
Trihelix-2B-1 49,642,780 9,375,668
TUB-1B-1 61,032,540 7,429,468
TUB-3A-1 52,739,064 17,779,513
Vrn1-5D-1 76,937,672 7,556,115
WRKY-1D-1 39,472,466 17,429,727
WRKY-1D-2 77,893,180 18,628,939
WRKY-6A-1 23,901,952 10,604,028

Antibodies

Halo: Promega, cat # G7281, lot # 0000384548

Peak calling parameters

MACS2 was used with parameters: "-c ${Halo} -f BAMPE -g 14271578887 --nomodel --nolambda" , FDR < 0.05 and P value < 1e-10.

Data quality

The peaks detected from samples introduced with Halo tag only were considered as non-specific bindings, and TF peaks overlapping with peaks detected from Halo samples were removed for subsequent analysis. Peak numbers are listed below:
AP2-DREB-1A-1 244,870
AP2-DREB-1B-1 228,748
AP2-DREB-1D-1 244,122
AP2-DREB-2D-1 239,133
AP2-RAV-4B-1 424,412
AP2-ERF-4B-2 5,098
AP2-ERF-5B-2 96,729
AP2-DREB-5B-3 135,566
AP2-ERF-6A-1 69,258
AP2-ERF-6D-1 35,523
bHLH-1A-1 11,892
bHLH-7D-2 224
bZIP-2A-3 129,202
bZIP-3A-1 175,818
bZIP-3A-2 113,502
bZIP-5B-3 56,164
bZIP-7A-1 1,495
C2C2-Dof-4A-1 259,937
C2C2-Dof-4D-1 243,497
C2H2-2A-1 230,160
C2H2-3A-1 125,633

EIL-4D-1 20,791
GARP-G2-2A-1 672,630
GARP-G2-2B-1 316,825
GARP-G2-4A-1 43,303
GARP-G2-4D-2 301,029
GARP-G2-5B-3 315,378
HB-HD-ZIP-2A-2 2,765
HB-HD-ZIP-6A-1 77,918
HB-HD-ZIP-6A-2 42,577
HB-HD-ZIP-6B-2 54,786
HB-PHD-6A-1 223,167
HSF-U 33,065
MYB-2A-2 2,863
MYB-7A-3 200,700
NAC-2D-6 1,567
NAC-3A-1 1,849
NAC-6A-1 263,558
NAC-6B-1 248,604
NAC-6D-1 158,055
NAC-7A-1 2,532
NAC-7D-2 611
SBP-7B-2 4,112
Trihelix-2A-1 187,258
Trihelix-2B-1 9,419
AP2-B 188
AP2-D 526
AP2-ERF-3A-1 304
AP2-ERF-5B-1 79
AP2-ERF-5D-1 27
AP2-ERF-6A-2 3,073
Q-5A-1 158
Q-5B-1 208
bHLH-3A-2 76
bHLH-5A-1 68
bHLH-6A-1 53
bHLH-7A-2 47
bHLH-7A-3 21
bHLH-U 39
PIF-5D-1 78
bZIP-2A-2 24
bZIP-5A-1 70
bZIP-6A-1 10
bZIP-A 255
bZIP-B 579
C2C2-Dof-2A-1 203
C2C2-Dof-2D-1 91
REF6-3D-1_ampDAP 24,509
GARP-ARR-6A-1 510
GNI-2D 354
GNI-U 346
MYB-1A-1 149
MYB-1A-2 158
MYB-2A-1 153
MYB-3A-1 135
MYB-3D-1 77
MYB-4A-1 180
MYB-4A-2 131
MYB-5A-1 399
MYB-5B-1 154
MYB-6D-1 596
MYB-7A-5 36
Tamyb10-3B 232
Tamyb10-3D 490
NAC-1D-1 39
NAC-2A-3 184
NAC-2A-4 329
NAC-3D-1 297
NAC-7A-2 195

NAC-7B-2 194
NAM-B1-6A-1 292
NAM-B1-6D-1 416
WRKY-1D-2 7,900
Alfin-4D-1 104
B3-2A-1 214
B3-2B-2 41
B3-4A-1 133
B3-4B-1 102
B3-4B-2 74
B3-5A-1 95
B3-5B-1 112
B3-5D-2 31
B3-6D-1 26
B3-6D-2 117
B3-7B-1 124
B3-7D-1 161
B3-ARF-2D-1 8
B3-ARF-3A-1 87
B3-ARF-3B-2 180
BES1-6A-1 42
BES1-A 70
BES1-B 134
BES1-D 73
bHLH-4B-1 132
bHLH-5A-4 43
PIF-5B-1 70
bZIP-D 287
C2-A 19
C2-B 17
C2-D 4
TdDof-3A 585
C2H2-3D-2 21
C2H2-4A-1 933
C2H2-5B-1 41
C2H2-6A-1 49
C2H2-6B-1 39
C2H2-6D-2 856
REF6-3D-1 4,342
C3H-1A-1 88
C3H-1D-2 29
C3H-3A-1 153
C3H-3A-2 19
C3H-7B-1 15
DBB-7A-1 221
DBP-1B-1 67
DBP-1D-1 52
GARP-ARR-7A-1 58
GARP-ARR-7A-3 55
GARP-G2-3B-1 71
GRAS-2A-1 19
GRAS-4B-2 115
Rht-4B-1 90
Rht-4D-1 143
GRF4-2A-1 26
GRF4-2B-1 30
GRF4-2D-1 38
GRF-6A-1 62
HB-BELL-4B-1 36
HB-BELL-5D-1 34
HB-HD-ZIP-2A-1 123
HB-HD-ZIP-2B-1 46
HB-WOX-A 11
HB-WOX-B 31
HB-WOX-D 8
LIM-7B-1 80
LIM-7D-1 46
LOB-1A-1 19

LOB-3A-1 61
LOB-3B-1 23
LOB-3D-1 61
LOB-3D-2 41
TaMOR-4B 467
TaMOR-4D 204
MADS1-lgy3-4A-1 70
MADS1-lgy3-4B-1 13
MADS1-lgy3-4D-1 29
MADS-M-4D-1 92
MADS-MIKC-5A-1 189
MADS-MIKC-7A-1 83
TaSEP3-7A 841
TaSEP3-7B 606
TaSEP3-7D 44
TaVRT2-7D 272
Vrn1-5D-1 361
NAC-2A-1 212
NF-YB-1B-1 81
PLATZ-A 75
PLATZ-B 69
PLATZ-D 49
RWP-RK-2D-1 146
IPA1-7B-1 113
SPL16-gw8-7B-1 167
SPL16-gw8-7D-1 319
Tify-5A-1 89
Tify-5B-1 52
Tify-6B-1 229
TUB-1B-1 74
TUB-3A-1 30
WRKY-1D-1 9
WRKY-6A-1 19

Software

fastp (version 0.20.0); Trim Galore (version 0.4.4); Burrows–Wheeler Aligner (version 0.7.17-r1188); MACS (version 2.2.6); MEME software toolkit (version 5.1.1); R package motifStack (version 1.34.0); R package universalmotif (version 1.4.0); BLASTN (version 2.9.0); Circos(version 0.69-8); MAFFT (version v7.149b); R Jalview (version 2.11.1.3)
